# Genome-wide association of yield traits in a nested association mapping population of barley reveals new gene diversity for future breeding

**DOI:** 10.1093/jxb/ery178

**Published:** 2018-05-15

**Authors:** Rajiv Sharma, Fulvia Draicchio, Hazel Bull, Paul Herzig, Andreas Maurer, Klaus Pillen, William T B Thomas, Andrew J Flavell

**Affiliations:** 1 University of Dundee at JHI, Invergowrie, Dundee, UK; 2 The James Hutton Institute, Invergowrie, Dundee, UK; 3 Martin-Luther-University Halle-Wittenberg, Halle/Saale, Germany

**Keywords:** Genome-wide association scans (GWAS), nested association mapping (NAM), quantitative trait locus hotspot (QTL hotspot), yield-related grain traits

## Abstract

To explore wild barley as a source of useful alleles for yield improvement in breeding, we have carried out a genome-wide association scan using the nested association mapping population HEB-25, which contains 25 diverse exotic barley genomes superimposed on an ~70% genetic background of cultivated barley. A total of 1420 HEB-25 lines were trialled for nine yield-related grain traits for 2 years in Germany and Scotland, with varying N fertilizer application. The phenotypic data were related to genotype scores for 5398 gene-based single nucleotide polymorphism (SNP) markers. A total of 96 quantitative trait locus (QTL) regions were identified across all measured traits, the majority of which co-localize with known major genes controlling flowering time (*Ppd-H2*, *HvCEN*, *HvGI*, *VRN-H1*, and *VRN-H3*) and spike morphology (*VRS3*, *VRS1*, *VRS4*, and *INT-C*) in barley. Fourteen QTL hotspots, with at least three traits coinciding, were also identified, several of which co-localize with barley orthologues of genes controlling grain dimensions in rice. Most of the allele effects are specific to geographical location and/or exotic parental genotype. This study shows the existence of beneficial alleles for yield-related traits in exotic barley germplasm and provides candidate alleles for future improvement of these traits by the breeder.

## Teaser

Discovering QTL hotspots for yield-related grain traits in a population developed from 25 diverse wild barley lines reveals new opportunities for improving cultivated barley.

## Introduction

During the past decades, improvements in breeding and agricultural practice have led to several-fold increases in the yields of many crop plants. However, by 2050, current yield improvement rates for the world’s major staple crops are estimated to be insufficient to feed the growing world population (Tilman *et al.*, 2011). The small grain cereals rice, wheat, barley, rye, and oat contribute ~50% of the world’s food supply (FAO stat). Barley is the fourth most important cereal crop and it serves as a model species for the temperate cereals as it can grow in disturbed habitats (Baum *et al.*, 2007) and it tolerates quite stressful conditions, including drought, high and low temperature, and salinity. It therefore holds the promise of improving yield by expanding the area under cultivation to marginal regions (Tester and Langridge, 2011).

Dramatic yield improvements have been achieved in small grain cereals via the introduction of dwarfing genes in the 1960s and 1970s (Hedden, 2003), which improved the capacity both to withstand lodging and to respond to nitrogen-based fertilizers. Additional improvements involved selection on flowering time genes to extend the growing period and thereby increase yield-bearing capacity (Borlaug, 1983). The last big improvement involved breeding for disease resistance, which has stabilized cereal yield against microbial pathogens (Tilman *et al.*, 2002).

Most genetic improvements in yield involved intercrossing existing high-performing germplasms. While this has been productive, it has resulted in genetic loss across the whole cultivated genome, and this loss has been severe at multiple loci. Recent genomic surveys comparing wild and domesticated barley germplasms revealed high diversity in the former and severe selective sweeps involving hundreds of genes in the latter, indicating that much of this diversity has been lost as a consequence of domestication (Sakai and Itoh, 2010; Huang *et al.*, 2012; Hufford *et al.*, 2012; Xu *et al.*, 2012; Russell *et al.*, 2016).

Wild barley (*Hordeum vulgare* ssp. spontaneum) is the ancestor of modern cultivar barley and the source of many of the alleles currently deployed in barley agriculture. The introduction of resistance loci against pathogenic fungi from the wild into the cultivated gene pool (Steffenson *et al.*, 2007) demonstrates that the wild barley gene pool contains potentially useful alleles that are currently not being used, but it is less clear that loci directly promoting increased yield are available for exploitation. Furthermore, one of the major reasons that wild diversity is not widely used for crop yield improvement is its linkage drag of unwanted wild characters that are difficult to work with in breeding programmes and mask the much rarer beneficial alleles. This has stimulated the development of advanced backcross (AB) populations derived from crosses between wild and cultivar germplasm, followed by backcrossing to the cultivar background and multiple selfing (Li *et al.*, 2005; von Korff *et al.*, 2006). Such experimental populations allow the discovery of quantitative trait loci (QTLs) and beneficial alleles derived from wild germplasm.

Grain yield is the key trait for the breeder, but understanding its genetics is difficult, due to its quantitative nature and complex inheritance, which often interacts with the environment. However, grain yield component traits such as thousand grain weight (TGW), grain number per ear (GPE), grain area (GA), grain length (GL), grain width (GW), and ear length (EL) are usually highly heritable. Genetic mapping studies have shown genomic regions associated with TGW, a trait that is important for malting quality as well as yield improvement in barley (Mather *et al.*, 1997; Igartua *et al.*, 2002; Walker *et al.*, 2013; Cu *et al.*, 2016).

Multiple major genes affecting developmental pathways are known to influence grain traits by modulating the grain-filling stages (Alqudah *et al.*, 2014). For barley, the *Photoperiod 1* (*Ppd-H1*) (Turner *et al.*, 2005) dominant allele accelerates progression towards flowering in wild and winter barleys, whereas *ppd-H1* delays flowering and maturity in spring barleys, where long days are required for better grain filling (Jones *et al.*, 2008). *HvCEN* (Comadran *et al.*, 2012), a homologue of the Arabidopsis gene *TFL1*, regulates the induction of flowering. The *FLORICAULA* (*FLO*) locus (2H at 107.36 cM), encodes an orthologue of the Arabidopsis *LEAFY* transcription factor which is a counterpart of FLORICAULA in Antirrhinum (Alvarez *et al.* 1992). Lastly, *HvGI*, the barley orthologue of the *GIGANTEA* flowering locus of Arabidopsis (Dunford *et al.*, 2005) is known to affect multiple developmental pathways including flowering, light signalling, circadian rhythm, and miRNA processing (Mishra and Panigrahi, 2015).

The development of high-throughput single nucleotide polymorphism (SNP) assays for barley, such as 9K iSelect (Comadran *et al.* 2012), and the recent sequencing of the barley genome increase opportunities to locate accurately the loci that control yield component traits for this crop at the genome-wide level (Waugh *et al.*, 2009; Mayer *et al.*, 2012; Mascher *et al.*, 2017). Pin-pointing such loci using genome-wide association scans (GWAS) of large genetically diverse populations has two main advantages over studies involving bi-parental mapping populations, namely the ability to access multiple gene alleles per locus and higher mapping resolution because the former populations carry many more recombination breakpoints in their history (Rafalski, 2010; Pasam and Sharma, 2014). Problems associated with GWAS include: (i) genetic substructuring of the germplasm which gives rise to high rates of false-positive associations; and (ii) low allele frequencies which confer low statistical power to associations.

Multiparent populations such as nested association mapping (NAM) populations combine advantages of both of the above population types (Yu *et al.*, 2008; Buckler *et al.*, 2009; McMullen *et al.*, 2009; Nice *et al.*, 2016). NAM populations typically contain multiple alleles, and rare alleles are enriched due to the mating design. This allows QTL mapping for wild-derived beneficial agronomic traits in barley (Nice *et al.*, 2017). The HEB-25 barley NAM population of 1420 lines derives from 25 wild barley accessions, each crossed with the cultivar Barke, then back-crossed to Barke, and subsequently selfed three times (i.e. BC_1_S_3_). HEB-25 was developed to detect the effects of exotic wild barley alleles in the elite genetic background (Maurer *et al.*, 2015). It has been utilized in several recent studies, leading to previously unknown QTL identification, and identification of exotic alleles at both known and unknown loci affecting plant development, flowering time, rust resistance, and salt stress (Castillo *et al.*, 2010; Schnaithmann *et al.*, 2014; Maurer *et al.*, 2015, 2016*a*; Saade *et al.*, 2016).

The main objective of the present study was to investigate yield and yield-related component traits in the HEB-25 population under differing eco-geographical and fertilizer environments. We have conducted large field trials of the HEB-25 population in Germany and Scotland, under varying nitrogen fertilizer treatments. The phenotypes of nine yield and yield component traits were scored and associations sought between these data and corresponding genotypes assayed across the population using 5398 genome-wide SNPs. A genomic prediction model was used to confirm the robustness of the GWAS peaks detected for each location, separating treatment effects that led us to identify both pan-population and family-specific exotic allele effects. Our analysis reveals 14 QTL hotspots distributed across the seven barley chromosomes and corresponding family-specific exotic allele effects, both positive and negative, for several yield component traits.

## Materials and methods

### Plant materials

The HEB-25 population of 1420 (BC_1_S_3_) NAM lines developed using 25 highly diverse wild barleys crossed with the German spring barley cultivar Barke was used in this study. The population development design has been explained previously (Maurer *et al.*, 2015).

### Field trials

The HEB-25 population was planted in two geographically diverse locations [Dundee, UK (JHI) (56°28'53.71''N; 3°6'35.17''W) and Halle, Germany (51°29'46.47''N; 11°59'41.81''E). All trials were sown in spring; the end of March in Halle and mid-April in Dundee.

Trialling was undertaken at both sites for two growing seasons (2014 and 2015) under two nitrogen (N) treatment levels (N0 and N1). For N0 (low N) there was 40 kg ha^–1^ and 30 kg ha^–1^ residual nitrogen available at Halle and 30 kg ha^–1^ and 60 kg ha^–1^ at Dundee in 2014 and 2015, respectively. N0 treatment received no additional fertilization. For N1 (high N) 100 kg N ha^–1^ was set as the optimum N level and additional N fertilizer was added to achieve this level. N1 treatment received extra N (calcium ammonium nitrate) applied as a 22:4:14 NPK compound mineral fertilizer at sowing (JHI) or at the shooting stage (Halle). A total of 60 kg ha^–1^ of N was applied at both sites in 2014 and for the 2015 sowing the additional N at Dundee was unchanged but the Halle rate was increased to 70 kg ha^–1^.

In Dundee, 1371 HEB-25 lines were sown, together with cv. Barke and the 25 *H. spontaneum* parents, in a modified augmented design type 2 (MAD-2) trial. The main plots of the MAD-2 trial consisted of a row of 13 test entries with cv. Concerto as the central main plot control. Twenty main plots were sown in a column and each treatment accounted for six such columns. In addition, 21 of the 120 main plots contained cultivars Barke and Scarlett as control subplots, which were allocated at random within the main plot. Plots in Dundee consisted of two rows, 40 seeds each, 2.0 m in length, 0.25 m between rows, and 0.75 m between plots.

A randomized complete block design was used in Halle for each treatment: 1536 plots of two rows of length 1.40 m, spacing of 0.20 m between rows, and 0.50 m between plots. There were 1420 trial entries, 26 parents (25 wild barleys and cv. Barke), and 90 repeated control cultivars (Barke, Marthe, Quench, and Scarlett). In Dundee, 1371 entries were sown, together with the 26 parents, in a modified augmented design type 2 as follows. The plots were sown in 20 rows by 78 columns, broken down into six adjacent blocks of 20 rows by 13 rows, each block containing a central seventh column of cv. Concerto as the main plot control. Each N treatment accounted for six such columns. In addition, 21 of the 120 main plots contained cvs Barke and Scarlett as control subplots, which were allocated at random within the main plot. Each Dundee plot was two rows, each 2 m long and 0.25 m apart with 40 seeds in each row, and plots were separated from each other by 0.5 m in each direction.

### Phenotyping of yield traits for NAM lines

Standard plot samples were taken from each plot, to derive yield-related grain traits, when the majority heads were ripe (DGS@ 91). Each standard plot sample at Dundee in 2014 consisted of a 25 cm section taken from the middle of both of the two rows. This provided more material than needed, and in 2015 it was reduced to five plants taken from the same section. At Halle, standard samples consisted of 10 ears, in both 2014 and 2015, and an additional harvest of the entire plots occurred at Halle only to derive an estimate of grain yield (YLD=seed weight per plot).

For each standard sample, the number of ears was recorded then the material was threshed with a laboratory thresher. The standard sample was weighed and the grains counted and imaged using a MARVIN Digital Grain Analyser (GTA Sensorik GmbH, Neubrandenburg, Germany). These data were used to estimate GL, GW, GA (=GW×GL), grain roundness (GR=GW/GL), GPE, and TGW. The MARVIN Analyser can also report the numbers of seeds that fall into different grain width and length fractions. At Dundee, grain width and length fractions (in 1 mm increments between 3 mm and 12 mm for GL and 0.5 mm increments between 1.5 mm and 6 mm for GW) were measured. These data were used to derive the grain stability traits: standard error of grain length (SE_GL) and standard error of grain width (SE_GW). Trait descriptions—years, treatment, and locations—are given in Supplementary Table S1 at *JXB* online. Raw data for all the above traits are deposited in the public data repository e!DAL (https://edal.ipk-gatersleben.de/;Sharma *et al.*, 2017).

### Genotyping of NAM lines

The Illumina iSelect 9K chip containing 7864 SNPs was used to genotype HEB-25 lines. After filtering for minor allele frequency (<10%), heterozygosity (<12.5%), and duplicates, 5398 informative SNPs were used in this study (Maurer *et al.*, 2017). To obtain SNP effects, an identity by state (IBS) approach was used, and NAM line genotypes were coded using Barke as a reference allele, with homozygous Barke, heterozygous exotic wild, and homozygous exotic wild genotypes coded as 0, 1, and 2, respectively.

### Phenotypic data analysis

Spatial (within-trial) variation for all traits was explored, using control plot scores, without obtaining any notable improvement, so spatial correction was not applied in this study. Summary statistics of the phenotypic data and ANOVA were generated using Genstat version 18 (Payne, 2009). Generalized heritability (*h*^2^) values were calculated by applying the VHERITABILITY procedure of Genstat version 18 (Cullis *et al.*, 2006) after a REML analysis of the data with genotypes and their interactions with sites, years, and nitrogen level set as random factors, and the main effects of site, year, and nitrogen fixed wherever present. As there was no replication, the interaction between genotypes, sites, years, and nitrogen levels was taken as the error.

### Genome-wide association and linkage disequilibrium analyses

To perform GWAS on yield traits, Model B (Liu *et al.*, 2011; Würschum *et al.*, 2012) was used as described by Maurer *et al.* (2016*b*). Besides a main effect for HEB family, Model B uses cofactors to control the genetic background and SNP effects are included as main effects using quantitative IBS genotype matrix scores [Model B, Y=μ+HEB family+∑SNP_IBS_+ε]. Cofactors that minimize the Schwarz Bayesian Criterion (Schwarz, 1978) were selected using ‘Proc GLMSELECT’ in SAS software (SAS Institute Inc.,Cary, NC, USA). During the stepwise forward–backwards selection procedure of cofactors, SNPs were allowed to enter or leave the model at each step until a further reduction of the Schwarz Bayesian Criterion was not obtained (Schwarz, 1978). To control false positives, a conservative threshold value of (–log_10_*P*=5) was used in GWAS to give consistent values for comparison across traits. To estimate the proportion of phenotypic variance explained by an SNP, *R*^2^ was obtained from a linear regression model.

Linkage disequilibrium (LD) between polymorphic SNPs (>5% minor allele frequency) was calculated using the solid spine method in Haploview 4.2 to define the extents of QTL intervals within the barley chromosomes (Supplementary Figs S13–S22). Where several significant SNPs were detected in a haplotype block, only the most significant was retained.

### Cross-validation

Five-fold cross-validation was run 20 times for each trait, for each location, year, and treatment combination. For this purpose, the total data set was divided into subsets containing 80% of randomly selected HEB lines per family and used for GWAS. The significant SNPs obtained therein were used to predict the phenotypic values of the remaining 20% of lines as a test set. The squared Pearson product–moment correlation (*R*^2^-value) was estimated between predicted and observed phenotypes in the test set. Finally, the detection rate (DR) was obtained as the number of times an SNP showed significance across the 100 cross-validation runs.

### Family-specific donor allele QTL effects

To estimate the exotic allele QTL effects among the 25 NAM families, we use the cumulated significant effects method described in Maurer *et al.* (2016*b*) which is based on Model A of Liu *et al.* (2011), where the family main effects are excluded. The procedure involved first the identification of a QTL peak marker that showed the highest detection rate across all cross-validation runs. Since the wild introgressions in HEB25 extend 26 cM on average (Maurer *et al.*, 2017), each peak marker was placed at the centre of a 26 cM interval and the SNP effects for each of the HEB-25 wild donors were estimated following the formula, $\sum_{i}^{n}SNP{\left(donor\right)}_{i}\times \alpha_{i}$, where *i* iterates through all significant SNPs (*n*) of the same size interval. Here SNP(donor)_*i*_ indicates the donor genotype (0 or 2) of the *i*th significant SNP and α_*i*_ indicates the SNP effect, obtained from the Model A GWAS analysis.

## Results

### Phenotypic analysis

**Fig. 1.**
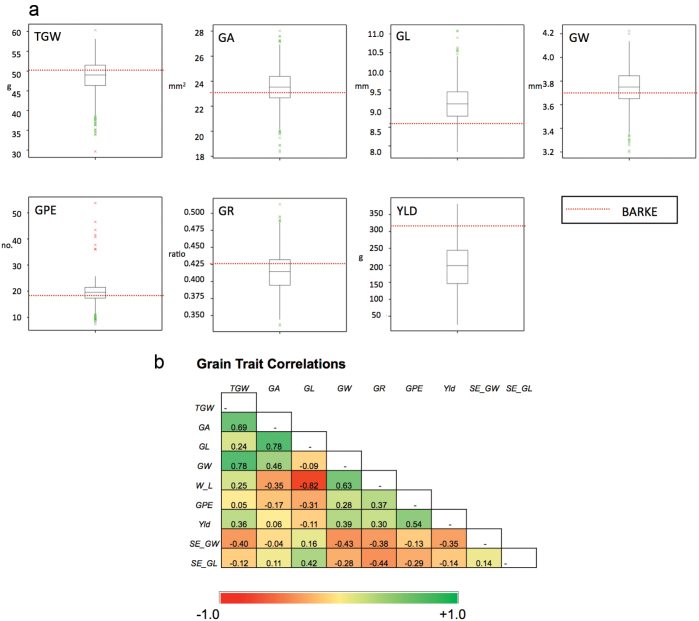
Phenotypic distribution of yield traits. (a) The box-plots display yield component traits based on averages over sites, locations, years, and treatments. The dotted red lines indicate the Barke (recurrent parent) trait scores. (b) Pearson’s correlation coefficients between yield component traits based on trait means across treatments, sites, and years. Abbreviations of yield component traits: thousand grain weight ‘TGW’, grain area ‘GA’, grain length ‘GL’, grain width ‘GW’, grain roundness ‘GR’, grains per ear ‘GPE’, ‘YLD’ yield. Green=positive correlation, red=negative correlation.

Field trials were performed for the complete HEB-25 population in Scotland and Germany over 2 years, with two different N fertilizer treatment regimes, occupying >12 000 plots in total. For all recorded grain yield traits, strong variation was observed within the population. Transgressive segregants surpassing the recurrent elite parent ‘Barke’ were observed for all yield-related traits (Fig. 1a). Coefficients of variation for the traits were in the region of 5% for GW, 10% for TGW, GA, GL, GR, and SE_GW, 20–34% for GPE and SE_GL, and 40–47% for GY (Supplementary Table S2). Moderate (>0.30) to high heritabilities (*h*^2^) were observed for all traits except GL in the N0 treatment at Dundee and SE_GW in both treatments at both sites, although both heritabilities were >0.30 across sites. The lower heritability of GL in Dundee N0 is probably due to threshability problems asscociated with the wild donor(s) that caused varying degrees of awn retention in the samples, which was exacerbated under the low N treatment. In general, higher values of *h*^2^ were found in Halle compared with Dundee, suggesting location and environmental differences (Supplementary Fig. S3–S11). ANOVA showed significant (*P*<0.001) genotype and N treatment effects for all traits at all locations (Supplementary Table S3). Genotype×year interactions were also significant for all traits except the two grain stability traits in Dundee and GA at Halle. In contrast, genotype×N treatment interactions were only highly significant (*P*<0.001) for GW and GPE at Dundee and never approached significance for any of the traits scored at Halle, suggesting a general absence of differential N treatment effects upon the genotypes studied.

Correlations among the yield-related traits are shown in Fig. 1b. TGW showed strong positive correlation with GA and GW, and weaker positive correlation with GL, GR, and YLD. TGW also showed a pronounced negative correlation with SE_GW. GA correlated positively and strongly with GL, GW, and TGW, and negatively with GR. GL showed an expected very strong negative correlation with GR but, interestingly, also a negative correlation with GPE. YLD showed positive correlations with TGW, GW, GR, and GPE, and negative correlations with GL and the stability traits SE_GW and SE_GL. At both locations, positive correlations were observed for all traits across N treatments (Supplementary Fig. S1; see diagonal). In general, lower correlations were observed at Dundee compared with Halle across N treatments, reflecting the occurrence of significant genotype×treatment effects in Dundee. In general, the correlations shown in Fig 1b and Supplementary Fig. S2 suggest that there is considerable independent genetic control for all the traits studied.

TGW did not show wide variations across site, year, and treatment combinations (Supplementary Fig. S3). In contrast, GA had a higher mean at the N0 treatment at both locations (Supplementary Fig. S4), but this was driven by a higher GL at Dundee (Supplementary Fig. S5) and a higher GW at Halle (Supplementary Fig. S6). Both GL and GW tended to be higher at Halle than at Dundee (Supplementary Figs S5, S6). Grain roundness (GR) varied with N treatments, but different trends were apparent between the two trial sites (Supplementary Fig. S7). GPE was consistently lower at Dundee compared with Halle (Fig. S8), and an opposite effect of N treatment was apparent between the two sites in 2014, with N1 being higher than N0 at Dundee. This may reflect differences in pre-anthesis growth conditions at the sites, with longer and cooler days at Dundee favouring an increase in fertile spikelets, whereas shorter and warmer days limited spikelet fertility at Halle in 2014. Surprisingly, YLD (scored only at Halle) was unaffected by increased N in 2014, but the expected yield increase was seen in 2015.

Variance components analysis revealed that the genetic main effect ranged from 5% (SE_GW) to 60% (YLD) of the total phenotypic variation (Supplementary Fig. S2). However, the sum of the genetic interactions was, with the exceptions of GL, GW, and SE_GW, less than the genetic main effect. The genotype×location effect was generally the greatest interaction, with the genotype×treatment component never being greater than 1%, confirming the ANOVA findings.

### Analysis procedure

We followed a tripartite approach to characterize the effects of the three major parameters under investigation here, namely wild donor germplasm type, genomic location, and environment, upon the grain yield traits: (i) GWAS analysis was performed on each of the 64 trait by trial (location/year/N treatment) combinations. Comparison of the QTLs identified across the trials yielded QTL hotspots (see below) wherein multiple traits coincide. (ii) To identify and locate genomic regions affecting yield grain trait differentially according to N treatment regime, cross-validation was run between the two N treatment regimes, using as inputs the combined data for the two trialling years at individual sites (i.e. Halle N0 2014 + 2015 versus Halle N0 2014 + 2015 and Dundee N0 2014 + 2015 versus Dundee N0 2014 + 2015). (iii) Family-specific donor effects were determined for each QTL to provide the contrasting allelic diversity values of the 25 wild barley donors.

### Genome-wide association of grain yield-related traits

GWAS analyses were performed separately for all 64 combinations of trait, treatment, year, and location (Supplementary Table S1). The Manhattan plots showed highly significant associations for all traits studied across the seven barley chromosomes (Supplementary Fig. S12). The most significant marker–trait associations for TGW, GA, GL, GW, GR, GPE, YLD, SE_GW, and SE_GL were located at chromosome 4H (14.9 cM), 1H (97.9 cM), 2H (55.5 cM), 4H (14.9 cM), 2H (56.2 cM), 2H (23 cM), 4H (3.5 cM), 3H (40.7 cM), and 1H (100.1 cM), respectively (Supplementary Fig. S12; Supplementary Table S4).

**Fig. 2.**
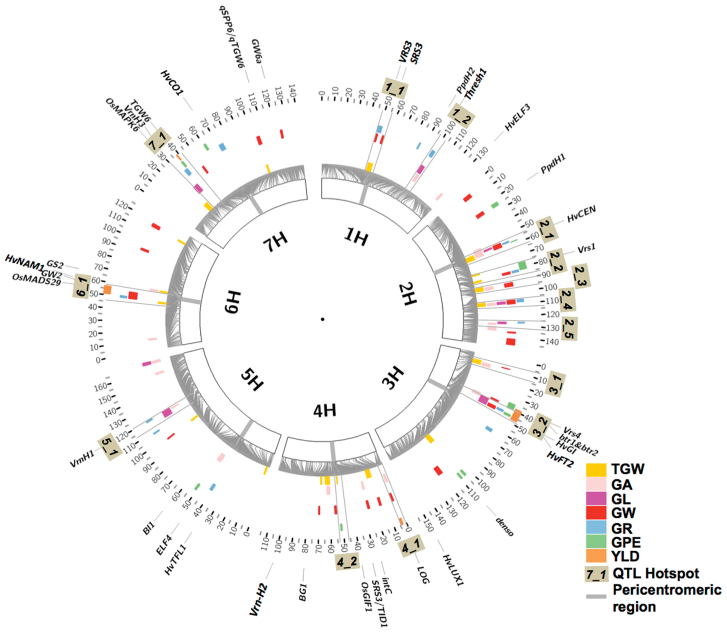
Circos graph displaying QTL hotspots for yield-related traits. QTL hotspot intervals, wherein several yield-related traits coincide, are bordered by grey radial lines. Map positions of candidate genes from barley and related cereal species which affect developmental and yield trait effects are also shown.

To discriminate QTL overlaps between trials, we used LD analysis of the iSelect SNP data (see the Materials and methods). This revealed 819 LD blocks distributed across the seven chromosomes, with a maximum of 172 on chromosome 5H and a minimum of 90 on chromosome 4H (Supplementary Fig. S13). This enabled us to identify 100 trait-specific QTL regions with significant (–log10_P_≥5) associations in at least three environments (Supplementary Figs S5–S11, S13–S22). These 100 QTL regions were then consolidated into 14 QTL hotspots containing QTL regions for at least three different traits (Fig. 2) (grain stability traits are not considered here due to low heritability, see below). Five of the QTL hotspots were found on chromosome 2H, with hotspot 2_1 showing significant effects for all yield-related traits except YLD. Every other chromosome contains between one and two QTL hotspots. Five hotspots are either located adjacent to or within the seven low-recombining peri-centromeric regions (Fig. 2; Supplementary Table S4). In addition, at least 10 hotspots also map close to known yield-related genes of barley (Fig. 2). The significance of these linkages is discussed later.

For the grain stability traits, we detected four QTLs for SE_GL that were significant in more than three environments; distributed on chromosome 1H, 2H, 3H, and 6H (Supplementary Fig. S22). Five QTLs for SE_GW were significant in more than three environments, three on chromosome 2H and one each from chromosome 3H and 4H (Supplementary Fig. S21).

The estimated phenotypic variation explained by SNPs across all measured traits was usually low, *r*^2^ <5% for 88% of SNPs. However, SNPs from genomic region 3H (44.8 cM) showed the highest explained variation for YLD (*r*^2^ up to 31%), followed by SE_GL (29%), GL (26%), and GR (22%) on chromosome 1H (100.0 cM) and GPE (23%) on chromosome 2H (76.2cM).

### Cross-validation analysis

To test the robustness of the GWAS-identified peaks and their sensitivity to N treatment effects, the genomic selection approach of Maurer *et al.* (2017) was used (see the Materials and methods). In total, 581 SNPs were robustly detected (DR ≥40) across the traits (Supplementary Table S5). A lower number of SNPs from Dundee (241) was significant compared with Halle (340). Almost equal SNP numbers were associated under N0 and N1 treatments (289 and 292, respectively).

**Fig. 3.**
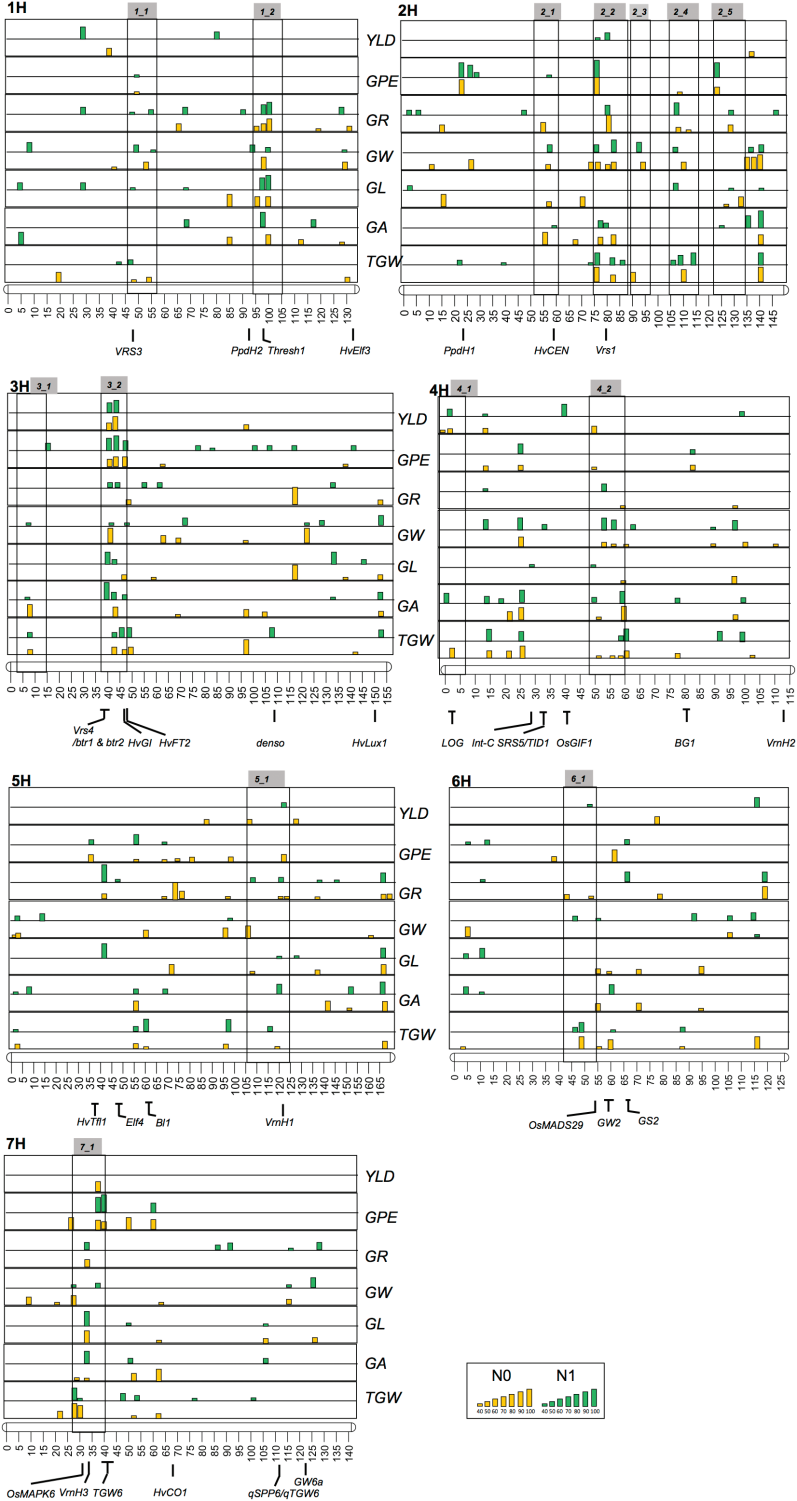
Genetic architecture of yield-related traits under varying nitrogen levels. Cross-validations (≥40) are displayed, where the height of the histogram bar corresponds to the number of cross-validations (see the Materials and methods). The highest significant SNPs are projected when multiple SNPs coincide over sites and N treatments (see Supplementary Table S5).

In total, these SNPs form 96 QTLs across all traits. Most of the significant regions were found across the N treatments, again displaying the relatively ineffective N treatment in our experiments (Fig. 3). Nevertheless, 31 QTLs were found to be significant to only one N treatment type (Supplementary Table S5). For example, QTLs on chromosome 1H, QTL_1H-3, QTL_1H-5, QTL_1H-7, QTL_1H-11, QTL_1H-15, QTL_1H-16, and QTL_1H-17, were present only in the N0 treatment. These N treatment-specific QTLs were dispersed on seven chromosomes of barley.

Seven QTL regions were only present in Dundee and 14 only in Halle (Supplementary Table S5). Fourteen, QTL hotspots discovered in GWAS analysis were also significant in the cross-validation across traits, N treatments, and locations (Fig. 3; Supplementary Table S5). Four of these hotspots were from the peri-centromeric low recombining region of the genome (QTL hotspots 1_1, 2_1, 4_2, and 6_1; Fig. 2; Supplementary Table S4–S6). However, five QTL hotspots appeared to show robustness for several traits and at higher cross-validation levels (>60). These were QTL hotspots: 1_2, 2_2, 3_2, 4_2, and 7_1, respectively (Fig. 3; Supplementary Table S5).

### Family-specific QTL effects

**Fig. 4.**
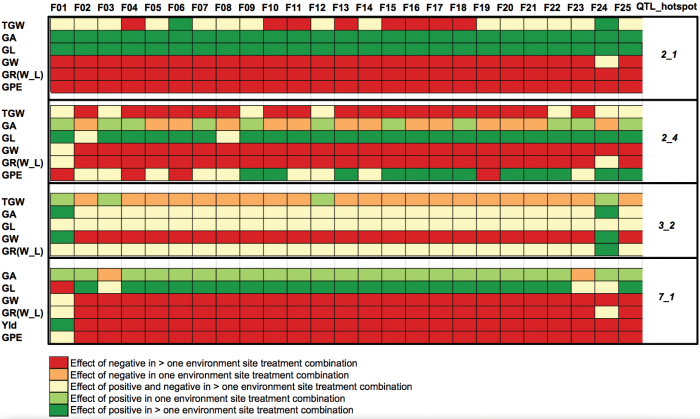
Family-specific effects at the four major QTL hotspots studied here. The cumulating method with a detection rate ≥25 was used (see Supplementary Table S6).

The 25 wild barley alleles contribute on average to increasing or decreasing trait values depending on the QTL. For example, the wild alleles at QTL hotspot 1_1 on average decrease the trait values of GR, GW, and yield stability traits, SE_GL and SE_GW; however, GPE and GL values are increased by wild alleles (Supplementary Table S5). Importantly, individual wild alleles from single families in several cases cause opposite effects of increasing and decreasing trait values, demonstrating the existence of both beneficial and harmful trait alleles within the HEB-25 population (Fig. 4). For example, 14 HEB-25 families are associated with decreasing GA and 11 with increasing GA values at QTL hotspot 2_4 (Supplementary Table S6). Within-family effects were largely similar, except 26 trait, treatment, and location combinations where the family effects were different across the families (indicated by a coefficient of variation >11 for the effects across families). The TGW QTL_3H_5 in Halle under N0 treatment is among the largest contrasting differences between HEB families. This region shows decreasing and increasing effects on TGW, varying between –3.04 g and +2.56 g depending upon the family (Supplementary Table S6).

## Discussion

A major goal for modern plant breeding is to create plant material that can withstand varying environmental challenges while delivering high yield. Yield *per se* is a complex end-product, deriving from many interacting developmental processes, at both the whole-plant and individual organ levels. This work seeks to find beneficial alleles from wild germplasm which control yield-related component traits defining the spatial dimensions of individual barley grains and the barley ear. We have also looked across different growth years, geographical locations, and N fertilizer inputs in order to probe the stability of these traits against environmental variation, and we measured the stability of the grains’ dimensions under these differing parameters.

We chose the HEB-25 NAM population for our study for two main reasons. First, it allowed us to interrogate 25 different wild barley genomes, providing a rich allelic diversity that should affect most of the major developmental processes that regulate barley grain yield under environmental change. Secondly, the BC_1_S_3_ genetic structure results in individual lines carrying ~25% wild germplasm against a background of ~75% cultivar genome, reducing the masking of beneficial trait effects by linkage drag from the wild genome. This choice has been justified by the strong QTL effects seen here and the identification of multiple QTL hotspots.

The phenotypic data collected here display large variation in the measured yield traits across the population. For all traits, we see transgressive segregants which surpass the trait values of the recurrent parental elite barley cultivar Barke, showing that wild alleles in our population can surpass a modern elite variety`s yield. We have also observed large genotype–environment interaction effects in our data set, particularly with regard to trial site. This is not surprising, since the two sites are separated by 5^o^ latitude, and Dundee has a maritime location whereas Halle is mid-continental, leading to pronounced differences in photoperiod and temperature change between the two locations across the seasons. For this reason, we evaluated our genome-wide scans separately for each combination of trait/location/year (see Supplementary Tables S2, S3; Supplementary Fig. S2).

Our approach has yielded 1065 SNPs (many of which overlap in their effects) that are associated at high significance with the trait data. To simplify this complexity and discover the most important genomic regions affecting the traits under investigation, we adopted a two-tier strategy to condense the 379 trait/environment/genomic location QTLs, first into 92 trait-specific QTL regions then into 14 QTL hotspots affecting at least three yield-related traits simultaneously (Fig. 2). Four of these (QTL hotspots 1_1, 2_1, 4_2, and 6_1) are in low recombining peri-centromeric regions, which hampers efforts to localize them accurately. However, using a candidate gene approach from related cereals, we propose candidate genes co-localizing to several regions (Supplementary Tables S4–S6; Supplementary Figs S3, S5–S13). In the next section, we discuss these four hotspots in detail.

### Major genomic hotspots for barley grain trait QTLs

#### QTL hotspot 2_1

All grain traits measured in this study except YLD coincide at this hotspot, which was also confirmed in the cross-validation analysis. A wild allele effect of +2.59 mm^2^ on GA (Halle N0) was seen within family 5 at hotspot 2_1 (Supplementary Tables S5, S6). This hotspot lies within the low recombining peri-centromeric region of chromosome 2H. This is a region of very high LD and thus contains many genes, but the obvious candidate gene in this region is *HvCEN* (Comadran *et al.*, 2012). Interestingly, this region also displays associations for almost all developmental phases studied by Maurer *et al.* (2016a) and has been picked up previously for yield and some component traits by Comadran *et al.* (2012) and Pasam and Sharma (2014).

#### QTL hotspot 2_4

As for hotspot 2_4, all grain traits except YLD are located in this region, which is close to the *FLORICAULA* (*FLO*) locus (107.36 cM) in a region of low LD (Supplementary Fig. S13). The rice *FLO* orthologue controls panicle initiation and could be the causative gene in this region. QTLs for ear length (Li *et al.*, 2005) and YLD (Saade *et al.*, 2016) have been mapped to this region previously, and 12 of the HEB parents contribute an increasing allele for GPE at this hotspot (Fig. 4).

#### QTL hotspot 3_2

Almost all yield-related traits showed overlap at this region (Fig. 2; Supplementary Table S5), which has low to moderate LD (Supplementary Fig. S13). This position has been picked up in other GWAS studies of yield (Nice *et al.*, 2017; Xu *et al.*, 2018). There are at least three candidate genes known to reside at or very close to this hotspot, namely the six-rowed spike 4 gene *Vrs4* (Koppolu *et al.*, 2013), the brittle rachis loci *btr1* and *btr2* (Pourkheirandish *et al.*, 2015), and *HvGI*, the barley orthologue of the *GIGANTEA* flowering locus of Arabidopsis (Dunford *et al.*, 2005). *Vrs4* was identified from six-row mutants with lateral spikelet fertility and loss of determinacy, but its role in natural variation of yield is not known. Brittleness, specified by the *btr* loci, has an indirect effect on yield (most of the seed falls to the ground before and during harvest) but *btr* alleles do not affect grain or ear dimensional parameters, whereas the QTL hotspot 3_2 does, so we conclude that *btr1* and *btr2* are not the major determinants of the QTLs found here. *Vrs4* controls the two-row/six-row switch via spikelet determinacy, thus strongly affecting all ear and grain dimension parameters. Lastly, *HvGI* is known to affect multiple developmental pathways including flowering, light signalling, circadian rhythm, and miRNA processing (Mishra and Panigrahi, 2015), so it is in our opinion an excellent candidate for this QTL hotspot. The wild parents of families 01 03, 12, and 24 contribute increasing TGW alleles at this hotspot, and families 01 and 24 in particular have broad positive effects upon individual grain component traits (Fig. 4).

#### QTL hotspot 7_1

This hotspot shows significance across almost all traits, the sole exception being GW that is significant only in Halle trials (Fig. 2; Supplementary Table S4). Cross-validation analysis revealed that the wild barley allele increases GA, GL, and SE_GW, whereas GPE, GR, YLD, and GW are reduced. All wild alleles at this hotspot, apart from family 1, reduce YLD and GPE. Almost all the wild parents contribute increasing alleles for GA at this hotspot, the exceptions being families 3 and 23. This appears to be due to an increase in GL that must be greater than the associated decrease in GW as the wild parents are generally contributing increasing alleles for the former and decreasing for the latter (Fig. 4).

The vernalization locus *VRN-H3* is located in this region (Yan *et al.*, 2006; Faure *et al.*, 2007; Turck *et al.*, 2008), and QTLs for YLD have been detected nearby in other AB-QTL studies (Li *et al.*, 2005; von Korff *et al.*, 2006). *VRN-H3* is the orthologue of the Arabidopsis *FT* gene that plays a central role in the flowering pathway, acting as a switch to progress from vegetative to reproductive growth under long-day conditions. Within the HEB-25 population, this region has been shown to be associated with multiple developmental traits including shooting, flowering and maturity, and plant height (Maurer *et al.*, 2016*a*), where wild alleles revealed a high diversity across the HEB families (Maurer *et al.*, 2017). Interestingly, SNP markers 4 cM away from *VRN-H3* have been found to be associated with reducing TGW (Maurer *et al.*, 2016*a*), but we observe GPE reduction in almost the same region. These two traits act inversely to each other in situations where GL is constant. Furthermore, we do not see significant correlation between these traits in our trials (see Fig. 1; Supplementary Fig. S1). This suggests that this genomic region contains a gene other than *VRN-H3* controlling GPE. Another possible source of this QTL is suggested by the region’s overlap with two rice grain trait genes, IAA-glucose hydrolase *TGW6* (Ishimaru *et al.* 2013) and a mitogen-activated protein kinase *OsMAPK6* (Liu *et al.* 2015) which influence grain size, TGW, and biomass. This is a region of low LD and constitutes a very credible candidate region for further high-resolution genetic studies to pinpoint the gene(s) underlying these traits.

### Other hotspots

We have identified other rice grain trait genes co-localizing at our QTL hotspots (e.g, *OsMAD29*, *GW2*, and *TGW6* at hotspot 6_1). These findings need future investigations to confirm whether the aforementioned candidate genes are causal agents for these QTL hotspots. Interestingly, previous studies in cereals also pointed to the existence of QTL hotspot regions (Marathi *et al.*, 2012; Rustgi *et al.*, 2013; Mikołajczak *et al.*, 2016; Wang *et al.*, 2016). Further, our QTL hotspot 2_2, which is genetically linked to the major barley row-type gene *VRS1*, corresponds to the QTL hotspot on chromosome 2H found in Wang *et al* (2016). We were able to identify this locus reliably although only a single family (family 24) shows the six-rowed phenotype. Furthermore, family-specific effects of increasing GPE and reducing TGW and GW in wild allele-carrying lines of HEB family 24 were observed, which are mainly due to the six-row spike morphology. It is interesting to see an overlap of the QTL hotspot 2_1 with regions E and F and of hotspot 4_1 with region I of the reported hotspots in the study of Mikołajczak *et al.* (2016). This locus has recently also been reported to carry a major flowering time QTL in the HEB-25 population affecting developmental and grain traits simultaneously (Maurer *et al.*, 2015, 2016*a*). Efforts are underway currently by us to clone the candidate gene behind the 4_1 region that also affects several developmental phases and grain traits. Moreover, Coventry *et al.* (2003) reported QTL localization in the bins 7, 8, 9, 10, 11, and 13, which most probably correspond to our QTL hotspots 2_1, 2_2, 2_3, 2_4, and 2_5 on chromsome 2H.

### Grain stability traits

The largest peak for the grain stability trait SE_GL is on chromosome 1H (100.1 cM) (Supplementary Tables S4, S5), with wild alleles increasing the parameter on average. Interestingly, the *thresh-1* locus (Schmalenbach *et al.*, 2011), controlling grain threshability, is linked to the SNP marker BOPA1 SNP marker 2_0267 BOPA1_1923_265 (Schmalenbach *et al.*, 2011), which maps to 98 cM and proved to be significant in our study. Poorly threshing lines are prone to overthreshing, which leads to seed damage and consequently variation in seed dimensions, in particular GL.

For SE_GW, QTLs on chromosomes 3H (40.7 cM) and 4H (14.9–26.3 cM) show high significance, with wild alleles contributing to an increase in the SE_GW. These regions coincide with the intermediate row-type loci, *int-C* and *Vrs4* (Ramsay *et al.*, 2011; Koppolu *et al.*, 2013), which both affect spike morphology and GW (see above).

### Environmental effects

With the increasing threat of global temperature rises, it is necessary to understand environment-specific effects at QTLs that control grain yield parameters. In our study, we provide an inventory of genomic regions which have large environmental effects. Several environment-specific effects on grain dimension have been found by us in HEB-25; in addition to the main effects on grain dimension discussed above (Supplementary Fig. S2; Supplementary Tables S4–S6), several environment-specific effects were also observed, as evident from the multienvironment analysis (Supplementary Fig. S2; Supplementary Tables S4–S6). For instance, N0-specific QTL effects from QTL_1H-12 and QTL_1H-16 appeared in Halle and QTL_3H-9 across locations. In several cases, candidate genes overlap with these QTLs. For example, *HvELF3* (Faure *et al*., 2012; Zakhrabekova *et al.*, (2012) maps to the QTL_1H-16 region. This gene affects the circadian clock and thus flowering, so it is not surprising to observe environment-specific effects on grain dimensions. Further investigation is needed to uncouple the effects of temperature and photoperiod, which often interact at several stages of plant development.

In this study, we aimed to uncover loci controlling grain yield component traits under varying N treatments. However, our trials failed to show major differences between the treatment levels. We have observed similar results previously for other barley germplasms (data not shown), and many of the trait values studied here tend to correlate between N treatments (Supplementary Fig. S1). The low rate of significant genotype by treatment interaction effects observed here (four out of 16, Supplementary Table S3) supports this interpretation.

We believe that one reason for this result is that residual N availability in our experimental fields, which were fertilized in previous years (see the Materials and methods), reduced the contrast between the two N treatment levels. Another possible explanation is that the HEB lines carry a high content of wild-derived, unadapted genome that renders them unable to respond to high fertility European environments.

### Family-specific effects: beneficial wild alleles

This study has revealed a number of strong family-specific allelic effects, which are contrasting between HEB families, suggesting high allelic diversity within the HEB-25 population (see above, Fig. 4; Supplementary Table S6). Several such allelic effects show improvements relative to the cultivar Barke allele (Supplementary Table S6), indicating that useful genetic diversity resides within the exotic gene pool. At the QTL_hotspot 2_1, exotic alleles from multiple HEB families, including F18, F24, and F25, are associated with up to a 6% increase over the cultivar Barke values for GA (Supplementary Table S6, column AN). Hotspot 3_2 also contains strong positive exotic allele effects, particularly from families 01 and 02, on GL, GR, and TGW, with no compensating deleterious effect on GPE (Supplementary Table S6, column AN). Furthermore, YLD improvements of up to 6.8% from the family 01 exotic allele, via improvement in both GA and GPE, are also associated with QTL hotspot 7_1, which suggests the relevance of the HEB-25 for yield improvements in barley. In comparison with bi-parental or unstructured diversity population analysis, our multiparental barley NAM design allows us to determine family-specific effects of wild barley alleles. This may guide future efforts in allele mining as we know the geographical locations of the wild barley donors of the HEB-25 population and therefore promising places to search for further trait-improving genetic diversity.

### Conclusion

An understanding of the genetic components underpinning yield-related traits is a pre-requisite to precision engineering of future yield improvement. We have used the genetic diversity of the HEB-25 NAM population to explore genetic loci specifying such traits in barley under varying environments. We find 14 QTL hotspots, at least 10 of which are located outside the low-recombining peri-centromeric regions, so are easily accessible for recombination-based breeding. Our analysis shows co-segregation of QTL hotspots with multiple developmental genes affecting flowering time and spike morphology. Our findings also highlight the importance of wild barley alleles in increasing TGW, but at the cost of reducing GPE and increasing height, with corresponding deleterious effects on grain yield. Balancing such positive and negative effects against each other to produce overall yield improvement will be the target for future breeding efforts. The wild-derived regions affecting grain size are associated with family-specific modulation in this parameter, and these regions can therefore be an entry point for future allele mining efforts. We are currently analysing exome-captured SNP sequence data of the HEB-25 population, which will allow us to discern family-specific haplotype effects in greater detail as a prelude to such activities.

## Supplementary data

Supplementary data are available at *JXB* online.

Fig. S1. Correlation matrix of the yield traits across locations and treatments.

Fig. S2. Variance components of the yield traits displayed in percentages.

Fig. S3. Box plot of thousand grain weight (TGW in g).

Fig. S4. Box plot of grain area (GA in mm^2^).

Fig. S5. Box plot of grain length (GL in mm).

Fig. S6. Box plot of grain width (GW in mm).

Fig. S7. Box plot of grain roundness (width to length ratio in %) (GR).

Fig. S8. Box plot of grains per ear (GPE in number).

Fig. S9. Box plot of yield (YLD in g, only from Halle).

Fig. S10. Box plot of standard error of grain width (SE_GW).

Fig. S11. Box plot of standard error of grain length (SE_GL only from Dundee).

Fig. S12. Genome-wide association scans of yield traits.

Fig. S13. LD of mapped SNP markers in the HEB-25 population.

Fig. S14. Distribution of thousand grain weight (TGW) QTLs across barley chromosomes.

Fig. S15. Distribution of grain area (GA) QTLs across barley chromosomes.

Fig. S16. Distribution of grain length (GL) QTLs across barley chromosomes.

Fig. S17. Distribution of grain width (GW) QTLs across barley chromosomes.

Fig. S18. Distribution of grains per ear (GPE) QTLs across barley chromosomes.

Fig. S19. Distribution of grain roundness (GR) QTLs across barley chromosomes.

Fig. S20. Distribution of grain yield (YLD) QTLs from Halle across barley chromosomes.

Fig. S21. Distribution of standard error of grain width (SE_GW) QTLs across barley chromosomes.

Fig. S22. Distribution of standard error of grain length (SE_GL) QTLs across barley chromosomes.

Table S1. Description of yield traits, locations, and years recorded.

Table S2. Summary statistics of yield-related traits.

Table S3. ANOVA results of phenotypic data within locations.

Table S4. GWAS results for grain traits.

Table S5. Cross-validation results for grain traits.

Table S6. Family-specific effect estimations based on the cumulating method.

Supplementary Tables S1-S9

Supplementary Figures S1-S22
