# Supplementary material for: Genome-wide association of yield traits in a nested association mapping population of barley reveals new gene diversity for future breeding
Source: J Exp Bot. 2018 May 15;69(16):3811–22. doi: 10.1093/jxb/ery178 (PMC6054221; doi:10.1093/jxb/ery178)
Supplement: Supplementary Figures S1-S22 [file ery178_suppl_supplementary_figures_s1-s22.pdf]

		N1							
		TGW	GA	GL	GW	GR	GPE	SE_GW	SE_GLn
Dundee N0	TGW	0.71	0.67	0.31	0.79	0.20	0.07	-0.24	-0.14
	GA	0.62	0.41	0.86	0.67	-0.41	0.13	0.00	-0.21
	GL	0.28	0.85	0.26	0.28	-0.78	0.09	0.08	-0.10
	GW	0.74	0.48	0.14	0.68	0.36	0.25	-0.10	-0.28
	W_L	0.14	-0.52	-0.83	0.42	0.45	0.04	-0.13	-0.05
	GPE	0.05	-0.06	-0.10	0.23	0.20	0.56	-0.03	-0.24
	SE_GW	-0.11	0.18	0.12	-0.12	-0.19	-0.11	0.57	0.12
	SE_GL	-0.07	0.10	0.26	-0.09	-0.26	-0.29	0.06	0.21

		TGW	GA	GL	GW	GR	GPE	YLD	SE_GW
Halle N0	TGW	0.79	0.60	0.17	0.75	0.22	0.02	0.37	-0.58
	GA	0.59	0.67	0.82	0.34	-0.49	-0.22	-0.01	-0.18
	GL	0.16	0.82	0.70	-0.18	-0.88	-0.31	-0.18	0.15
	GW	0.77	0.33	-0.15	0.83	0.62	0.21	0.38	-0.62
	W_L	0.24	-0.51	-0.88	0.59	0.82	0.34	0.31	-0.43
	GPE	0.09	-0.20	-0.31	0.28	0.38	0.87	0.45	-0.07
	YLD	0.37	-0.03	-0.18	0.36	0.31	0.49	0.80	-0.29
	SE_GW	-0.60	-0.18	0.17	-0.61	-0.43	-0.13	-0.32	0.48

-1.0	+1.0

Figure S1: Correlation matrix of the yield traits across locations and treatments. Red = negative correlation, green = positive correlation. Correlations between the treatments are shown diagonally.

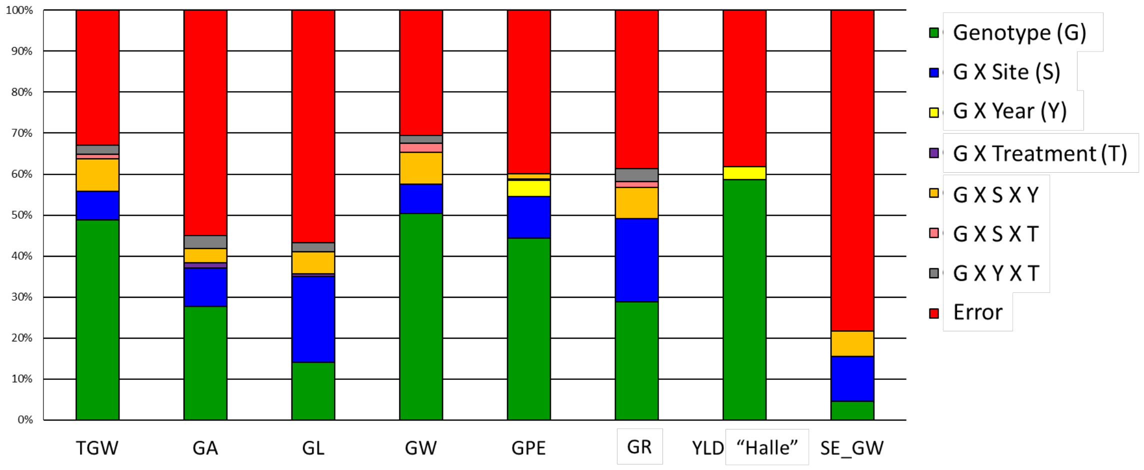

Figure S2: Variance components of the yield traits displayed in percentages.

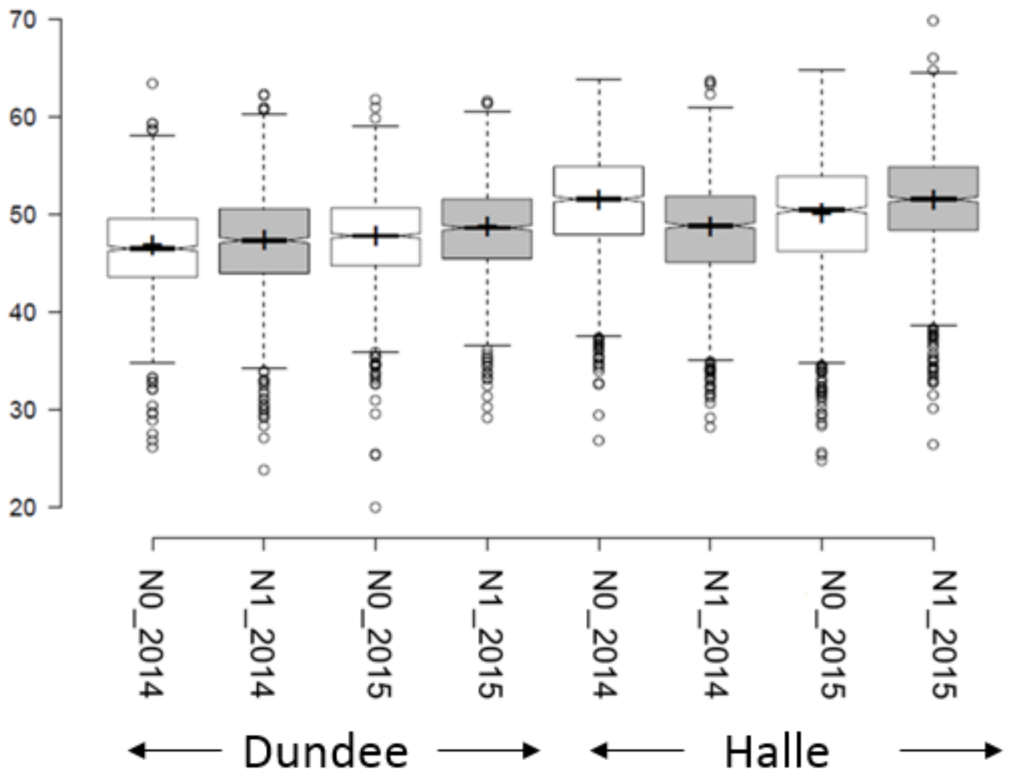

Figure S3: Box plot of thousand grain weight (TGW in grams).

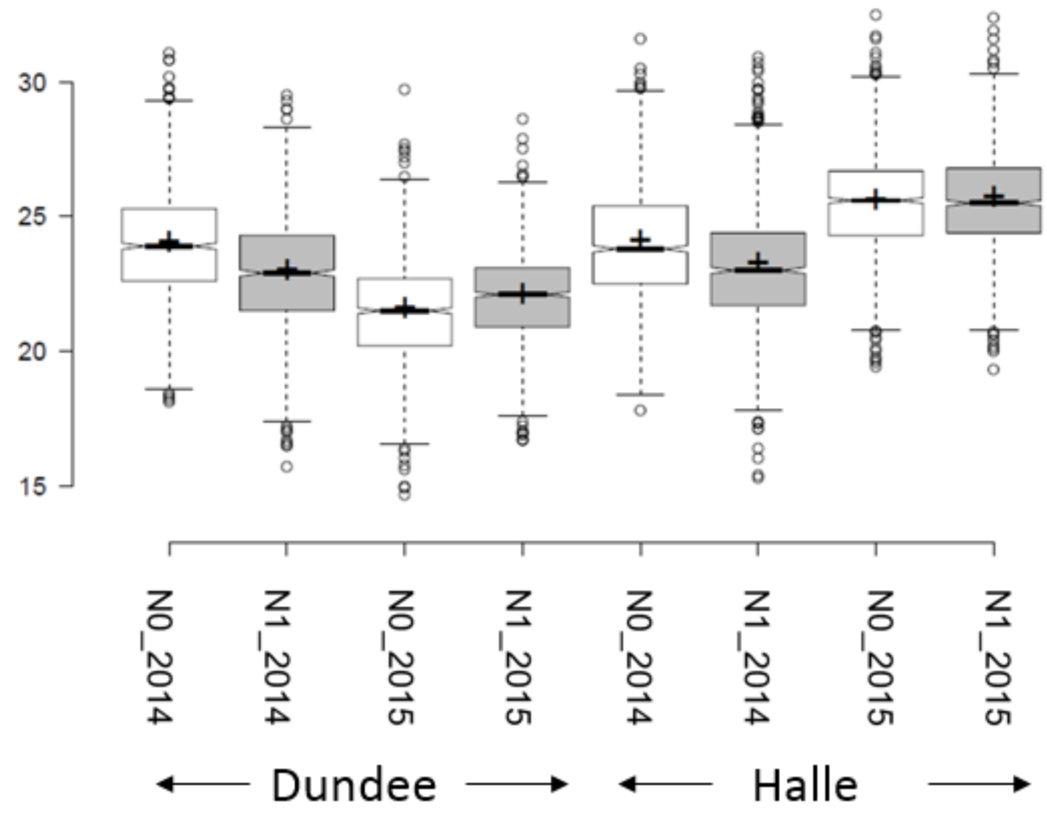

Figure S4: Box plot of grain area (GA in mm^2).

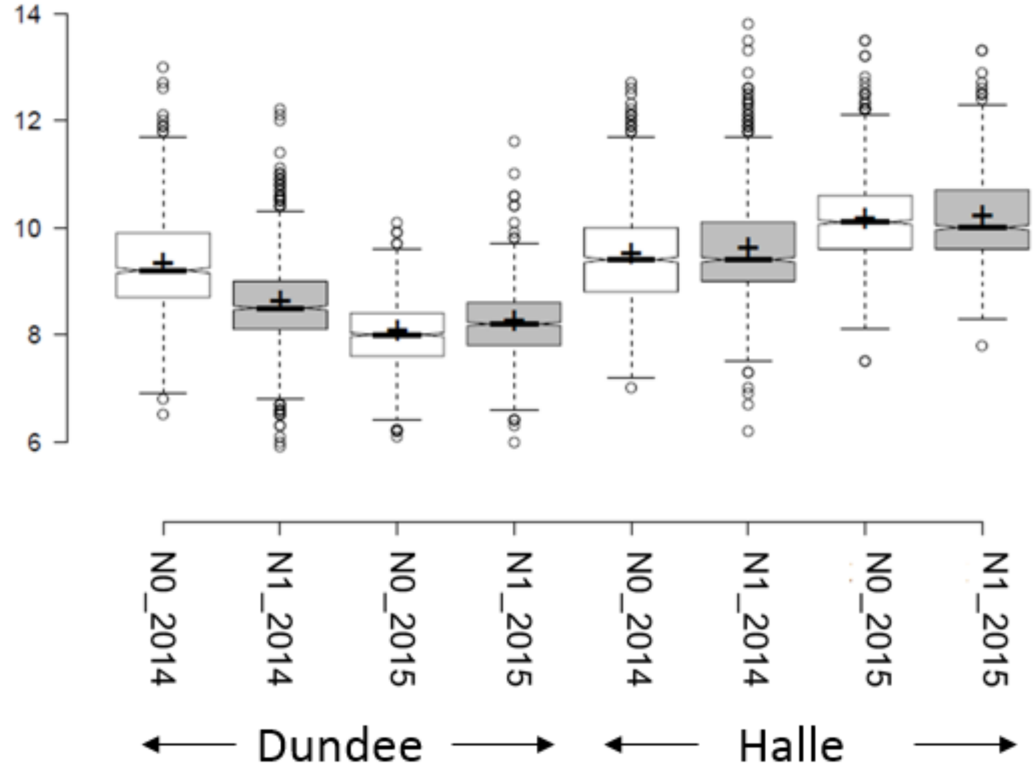

Figure S5: **Box plot of grain length (GL in mm).**

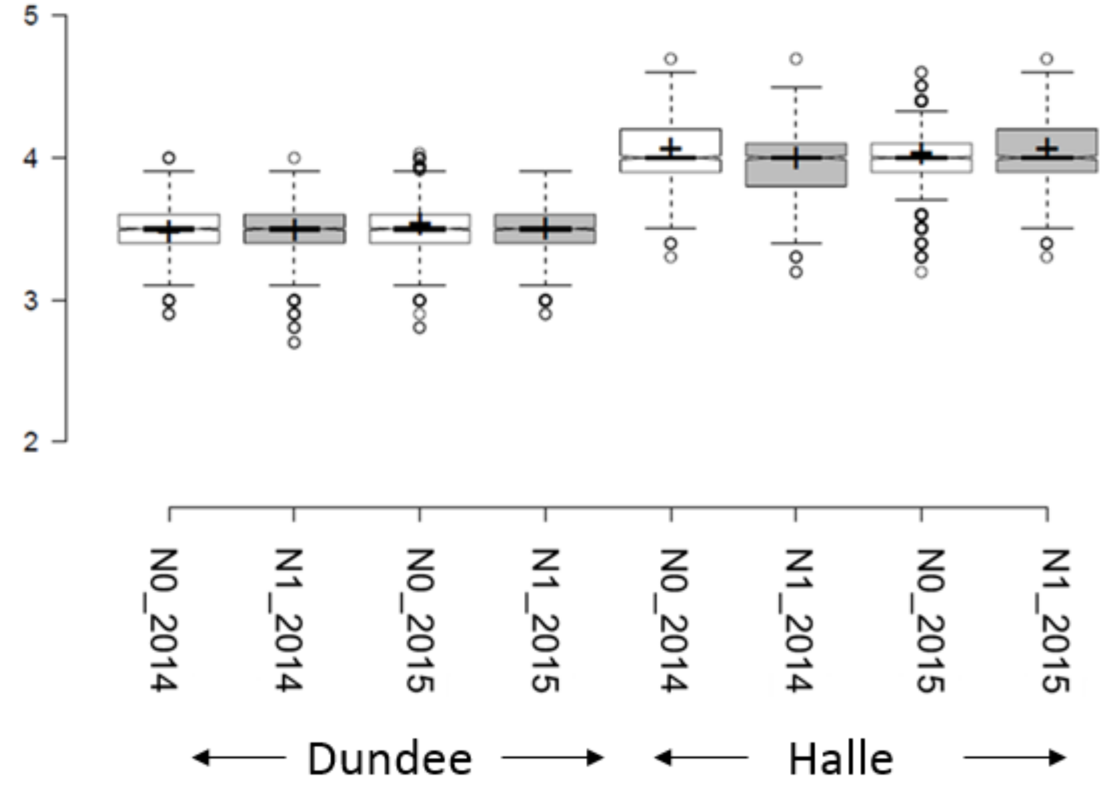

Figure S6: **Box plot of grain width (GW in mm).**

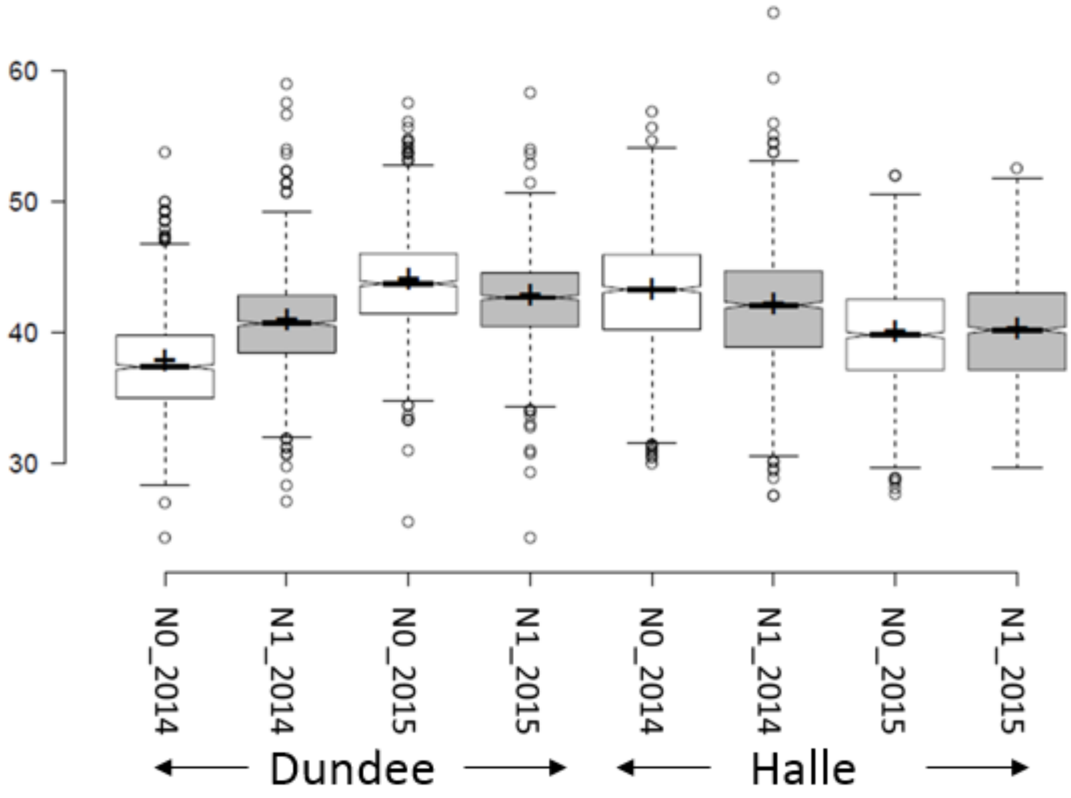

Figure S7: **Box plot of grain roundness (width and grain length ratio in %) (GR).**

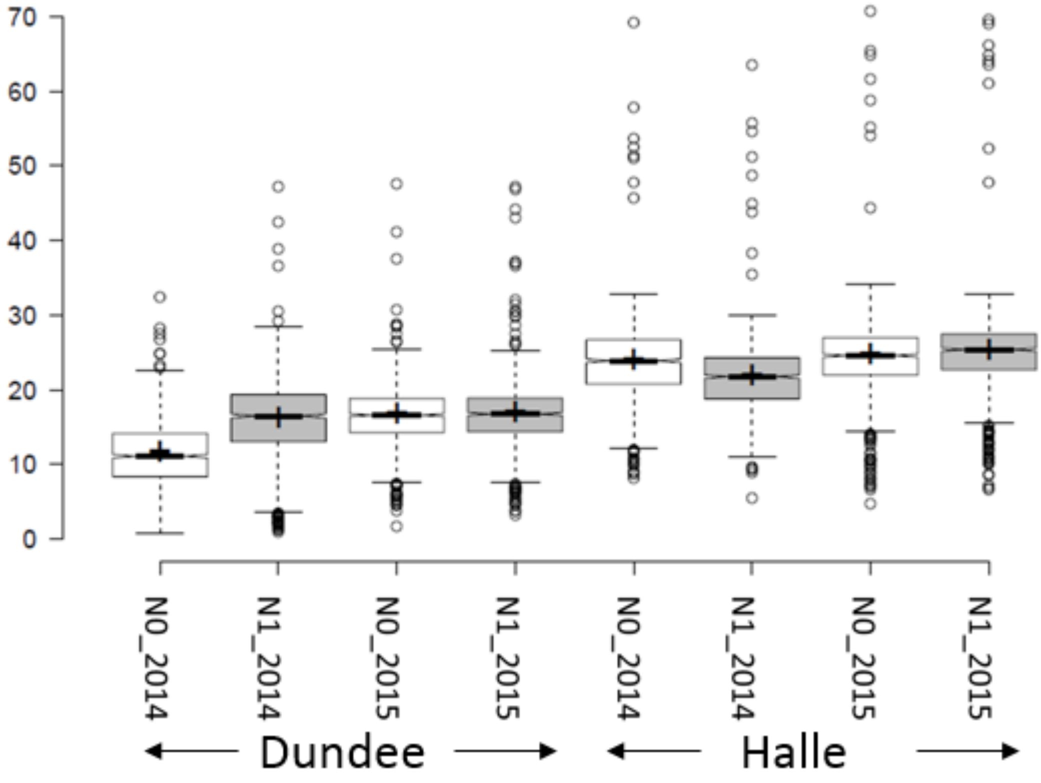

Figure S8: **Box plot of grains per ear (GPE in number).**

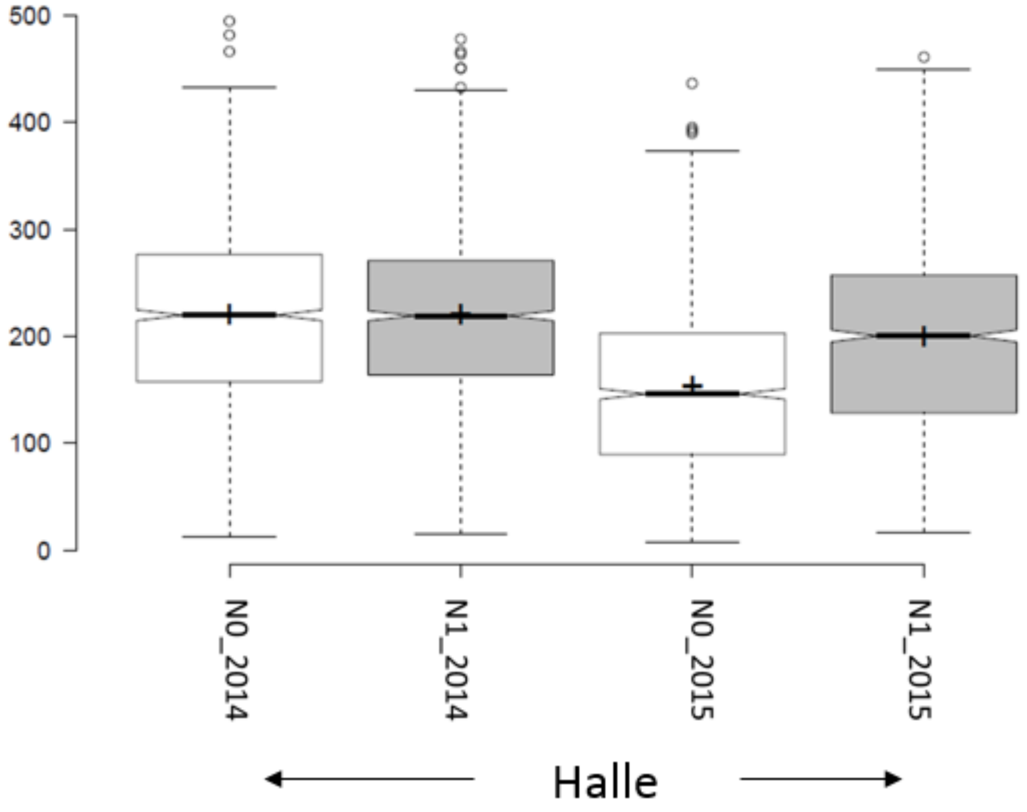

Figure S9: **Box plot of yield (YLD in grams, only from Halle).**

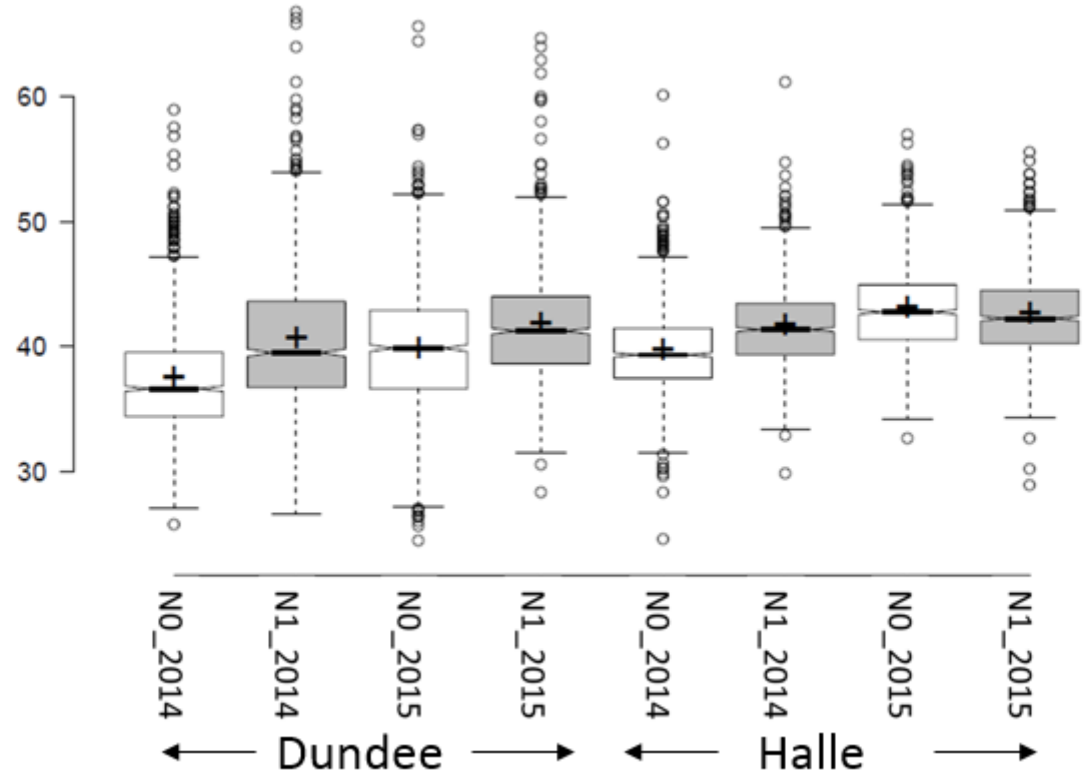

Figure S10: Box plot of standard error of grain width (SE_GW). To ease displaying, values are multiplied by 10.

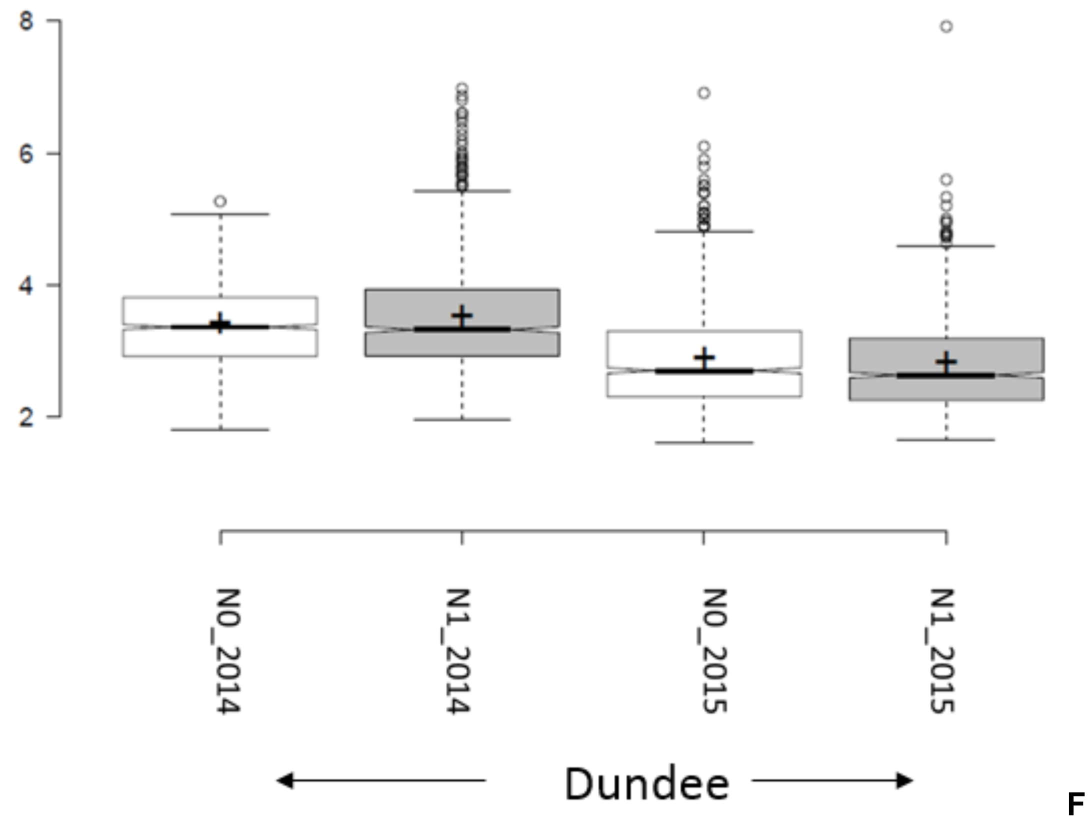

Figure S11: Box plot of standard error of grain length (SE_GL only from Dundee). To ease displaying, values are multiplied by 10.

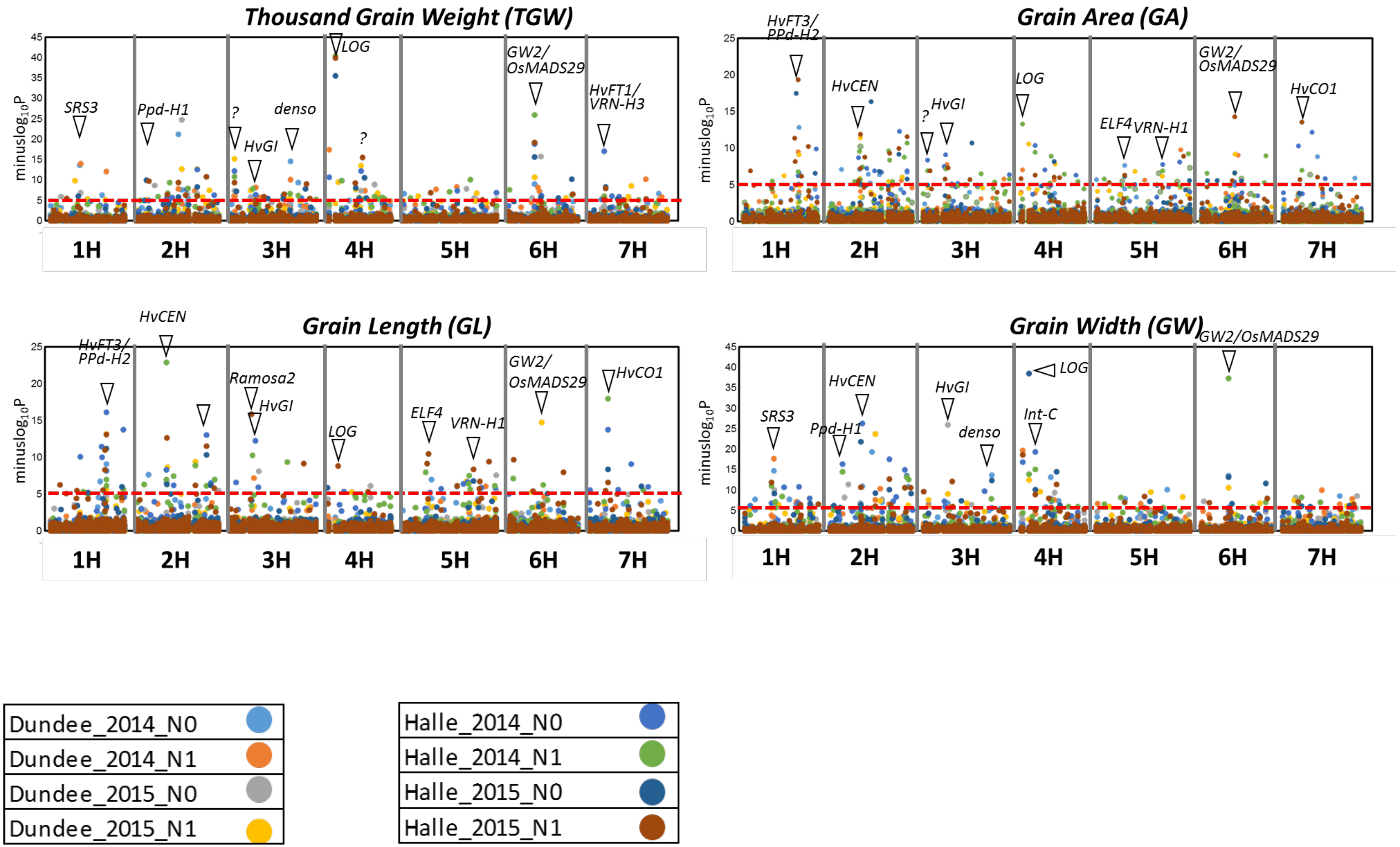
Thousand Grain Weight (TGW)
minuslog10P
SRS3
Ppd-H1
?
HvGI
denso
LOG
?
GW2/
OsMADS29
HvFT1/
VRN-H3
1H
2H
3H
4H
5H
6H
7H
Grain Area (GA)
HvFT3/
PPd-H2
HvCEN
?
HvGI
LOG
ELF4
VRN-H1
GW2/
OsMADS29
HvCO1
Grain Length (GL)
HvCEN
HvFT3/
PPd-H2
Ramosa2
HvGI
LOG
ELF4
VRN-H1
GW2/
OsMADS29
HvCO1
Grain Width (GW)
GW2/OsMADS29
SRS3
Ppd-H1
HvCEN
HvGI
denso
LOG
Int-C
Dundee_2014_N0
Dundee_2014_N1
Dundee_2015_N0
Dundee_2015_N1
Halle_2014_N0
Halle_2014_N1
Halle_2015_N0
Halle_2015_N1

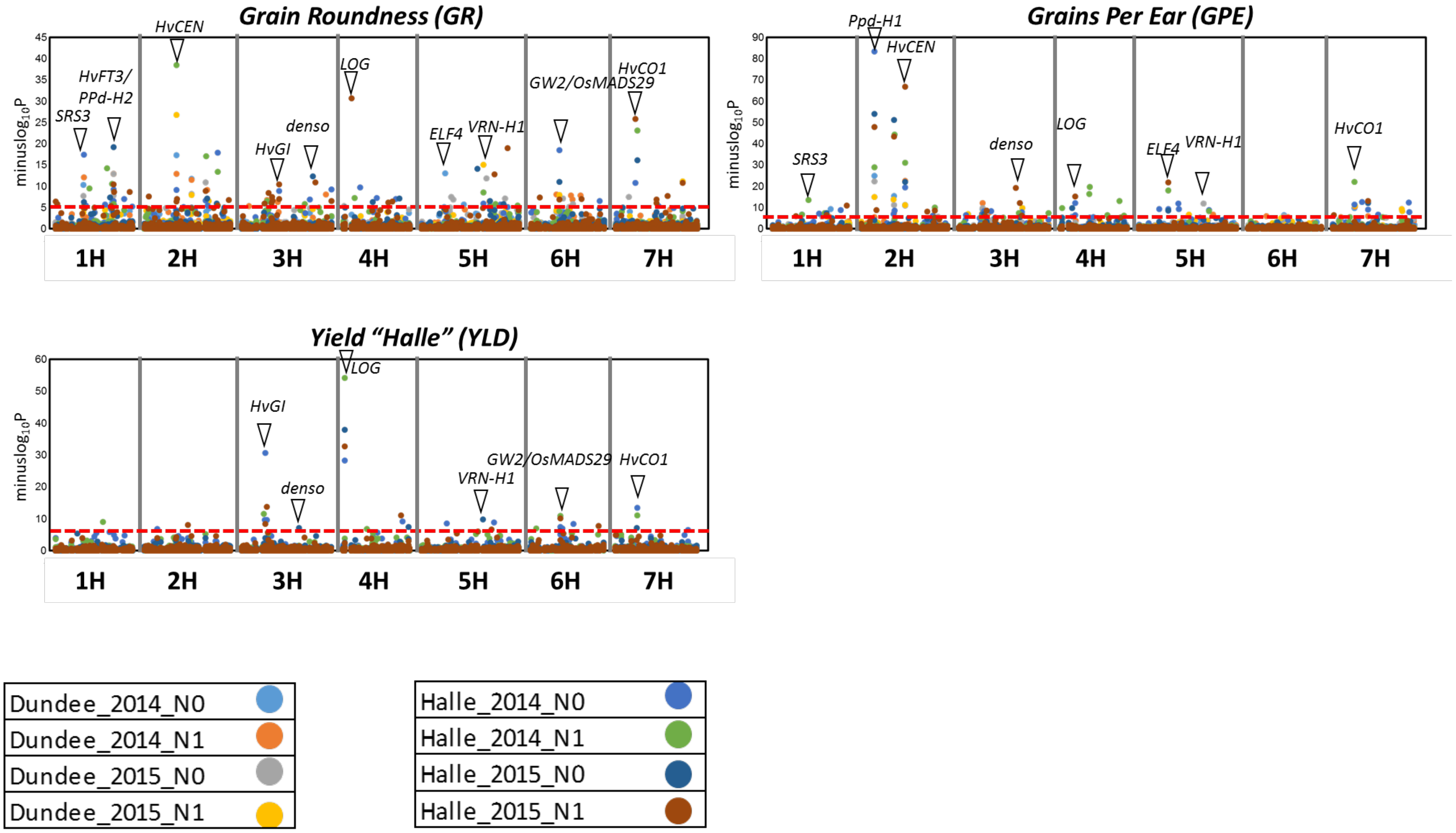

Grain Roundness (GR)
minuslog10P
45
40
35
30
25
20
15
10
5
0
HvCEN
HvFT3/
PPd-H2
SRS3
HvGI
denso
LOG
ELF4
VRN-H1
GW2/OsMADS29
HvCO1
1H
2H
3H
4H
5H
6H
7H
Grains Per Ear (GPE)
minuslog10P
90
80
70
60
50
40
30
20
10
0
Ppd-H1
HvCEN
SRS3
denso
LOG
ELF4
VRN-H1
HvCO1
1H
2H
3H
4H
5H
6H
7H
Yield "Halle" (YLD)
minuslog10P
60
50
40
30
20
10
0
LOG
HvGI
denso
VRN-H1
GW2/OsMADS29
HvCO1
1H
2H
3H
4H
5H
6H
7H
Dundee_2014_N0
Dundee_2014_N1
Dundee_2015_N0
Dundee_2015_N1
Halle_2014_N0
Halle_2014_N1
Halle_2015_N0
Halle_2015_N1

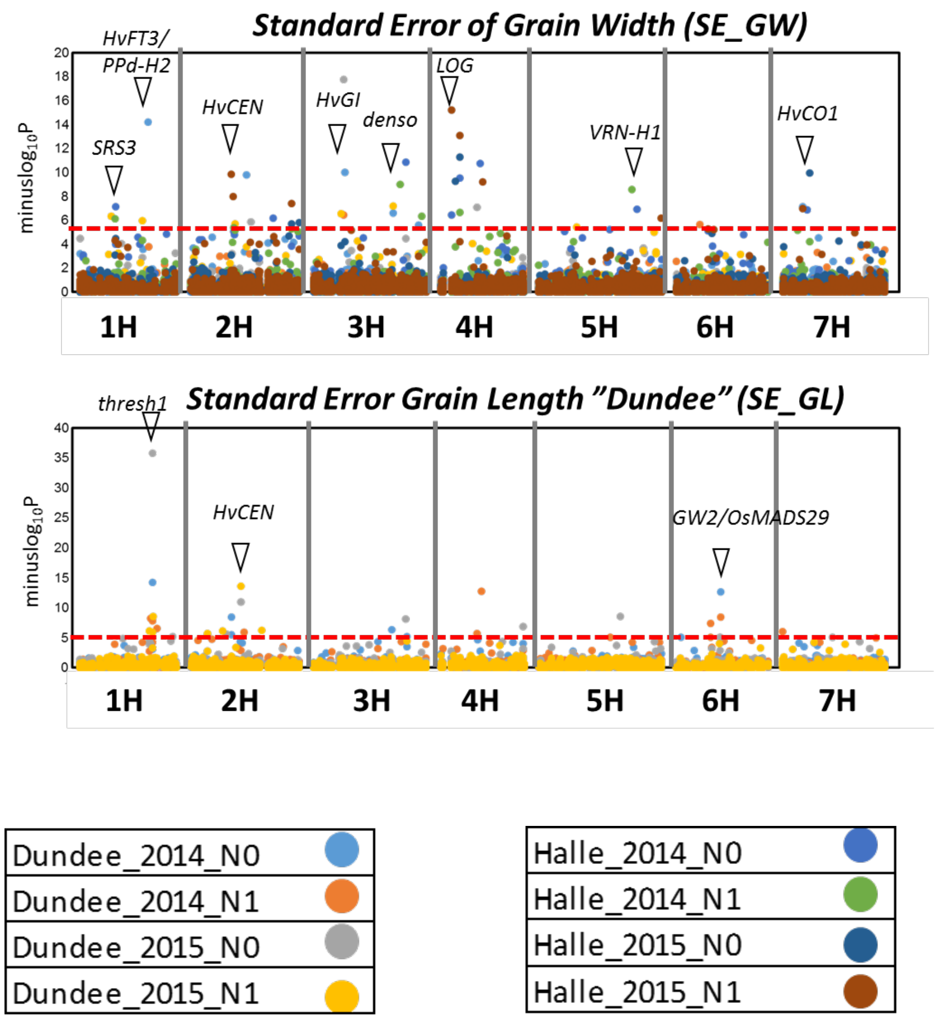

Figure S12: Genome-wide association scans of yield traits.

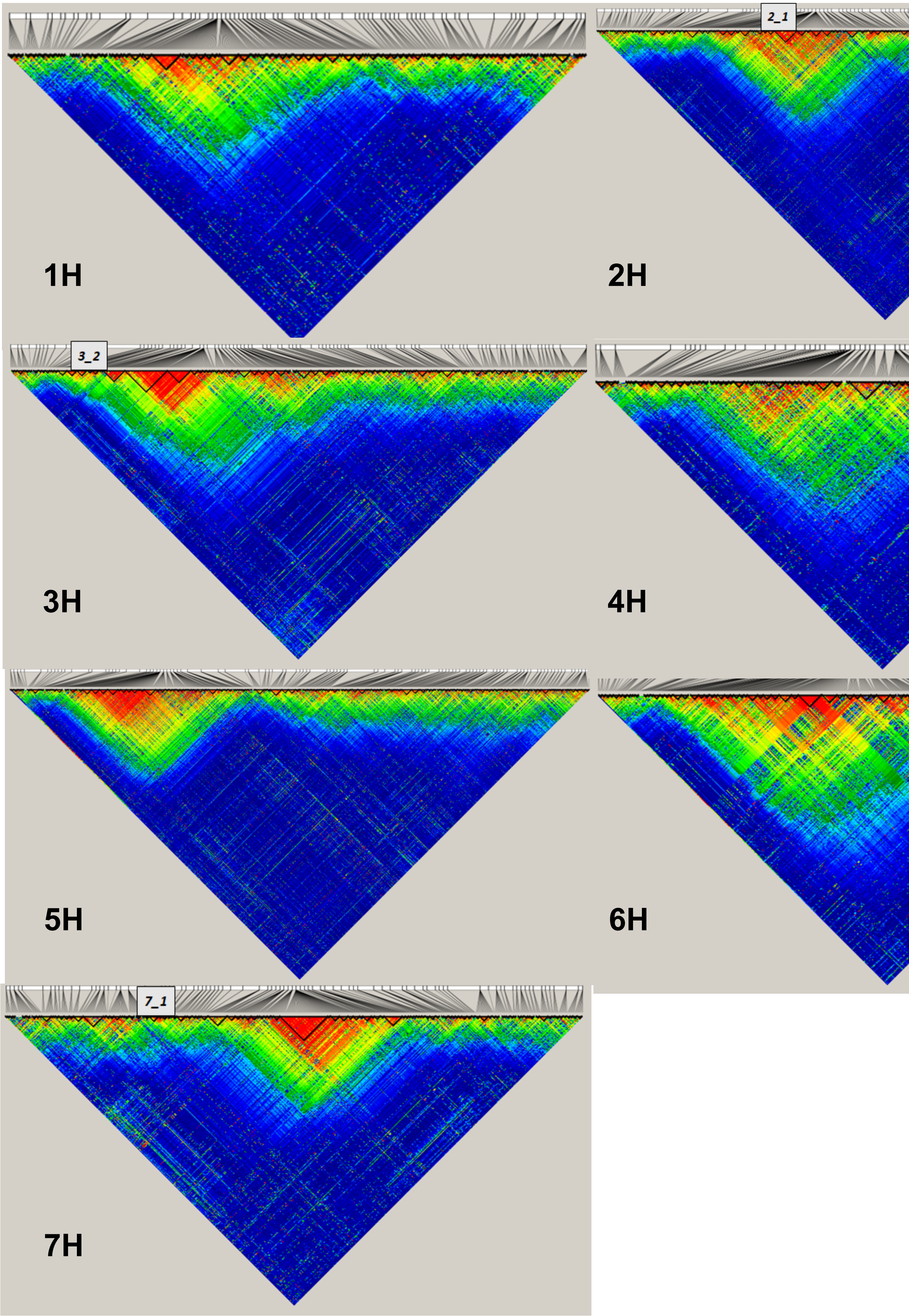
2_1
3_2
7_1
1H
2H
3H
4H
5H
6H
7H

Figure S13: LD of mapped SNP markers in HEB-25 population. The plot uses a GOLD heatmap color scheme implimented in Haploview 4.2. Blocks of LD based on the Solidspine alogarithm of Haploview4.2 are indicated as black outlines. The four QTL-hotspots discussed here are shown on chromsomes 2H, 3H and 7H.

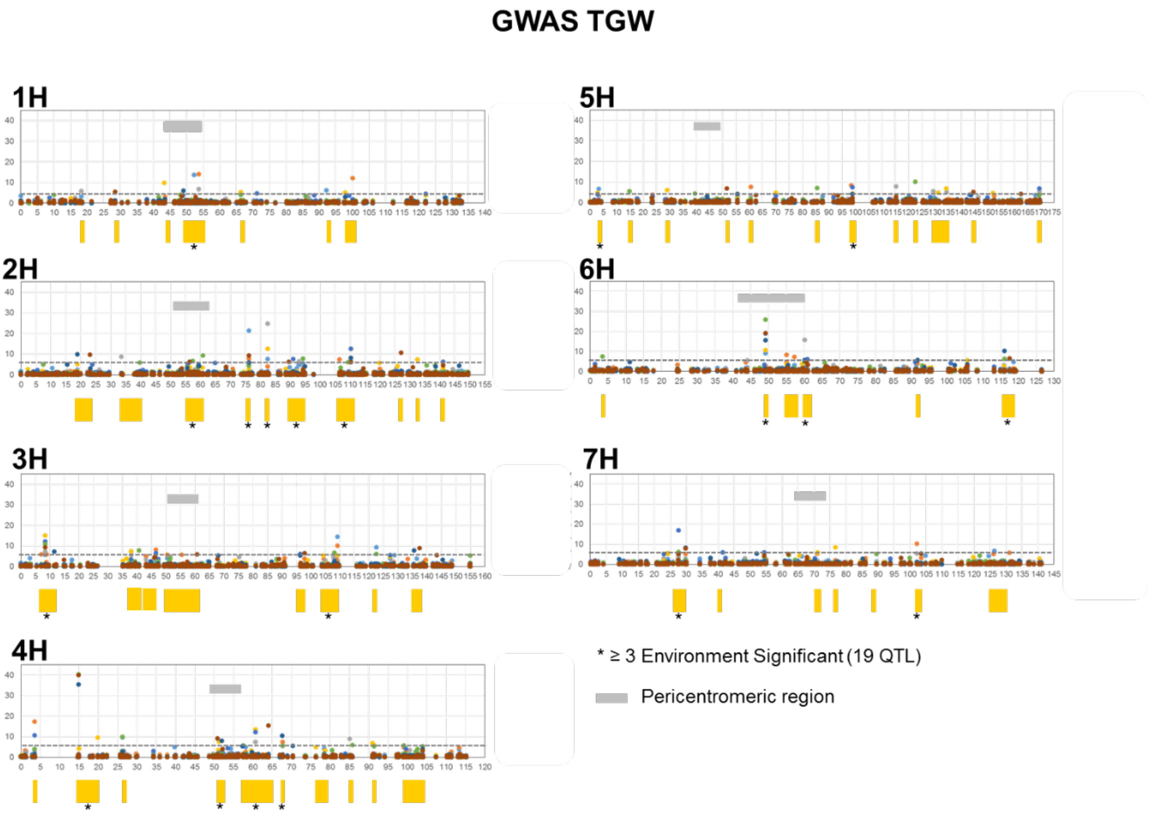

Figure S14: Distribution of thousand grain weight (TGW) QTLs across barley chromosomes.

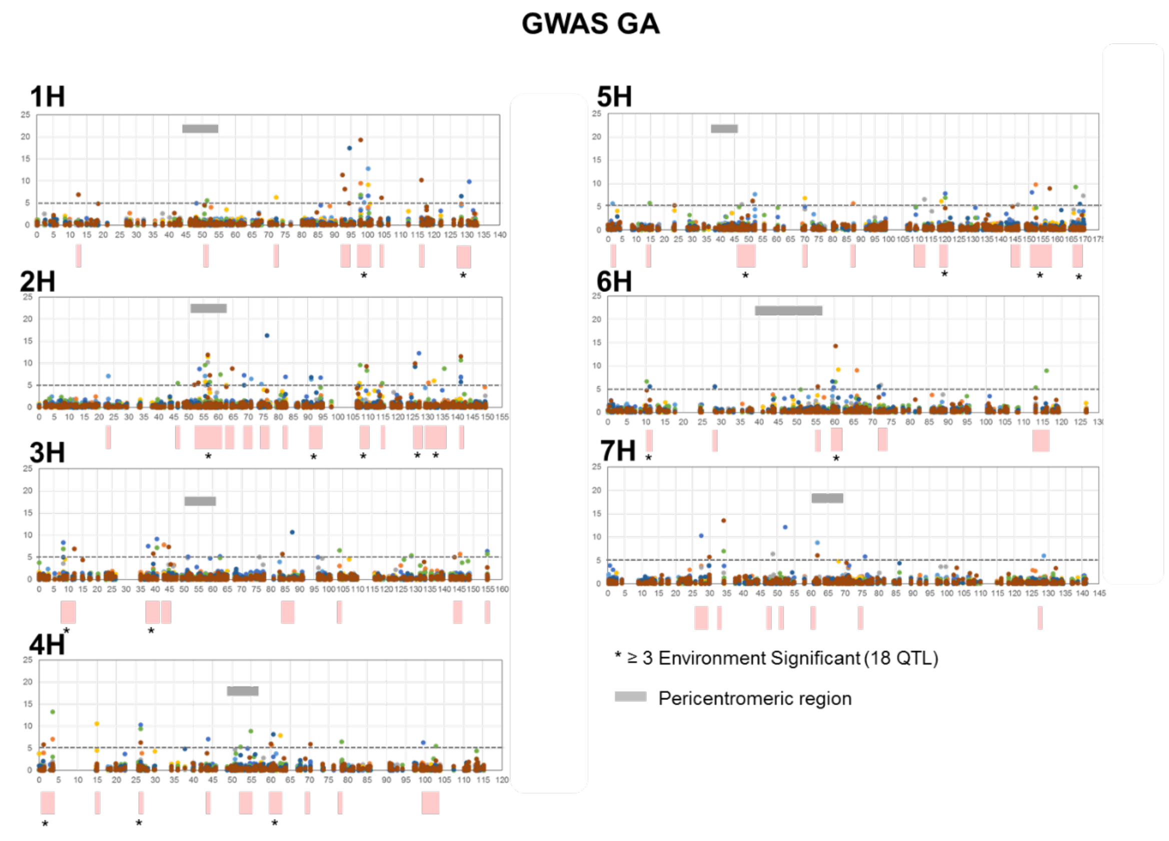

Figure S15: Distribution of grain area (GA) QTLs across barley chromosomes.

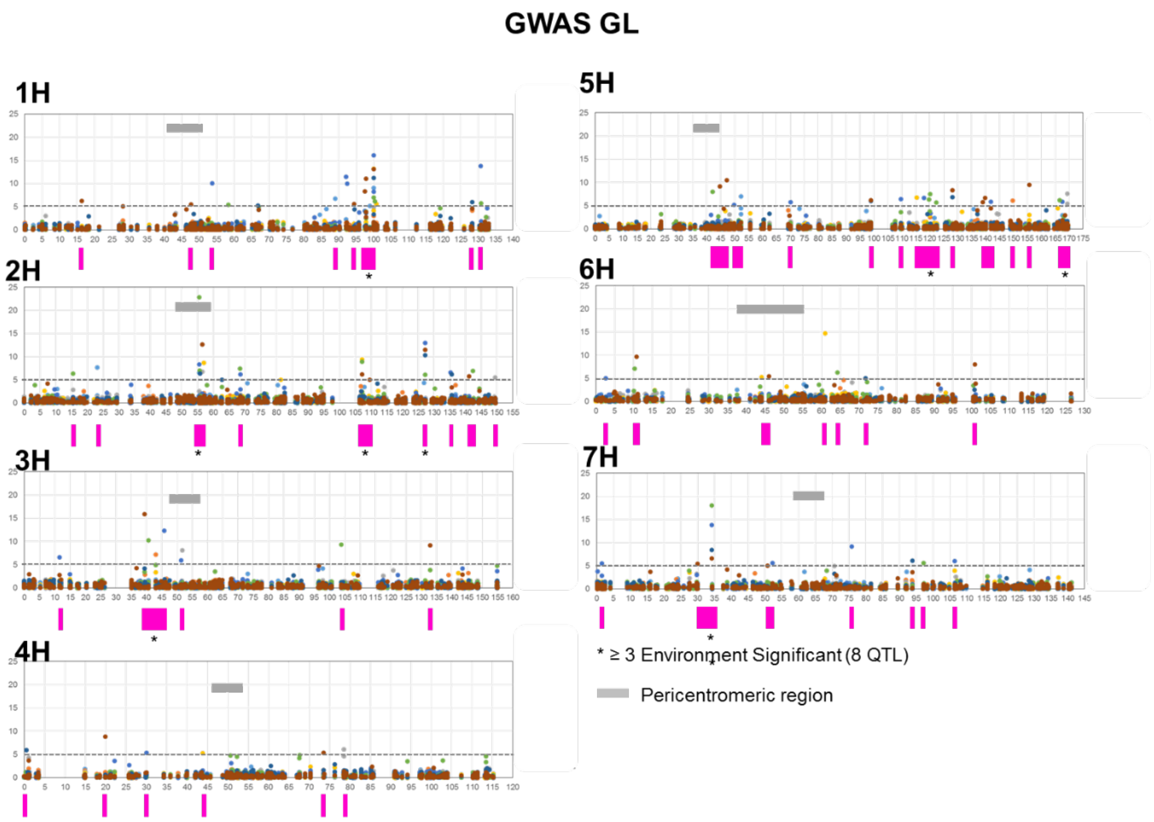

Figure S16: Distribution of grain length (GL) QTLs across barley chromosomes.

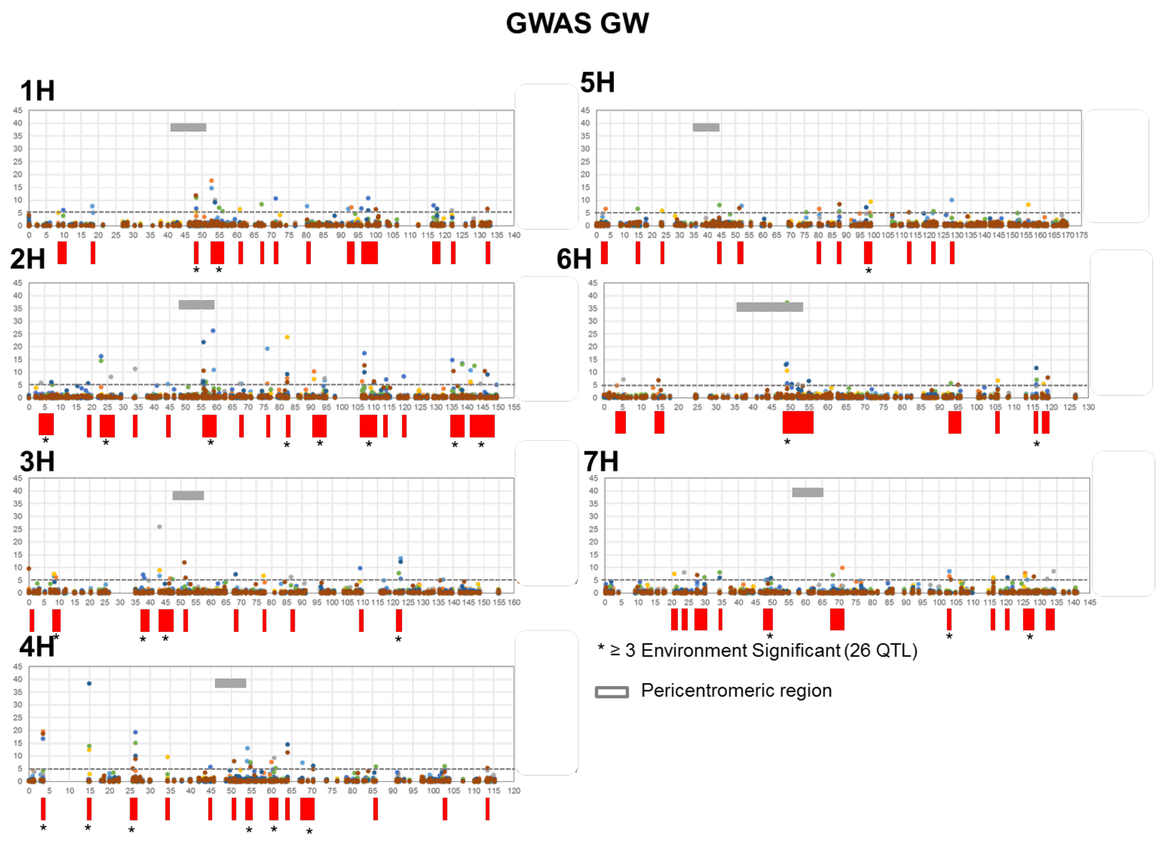

Figure S17: Distribution of grain width (GW) QTLs across barley chromosomes.

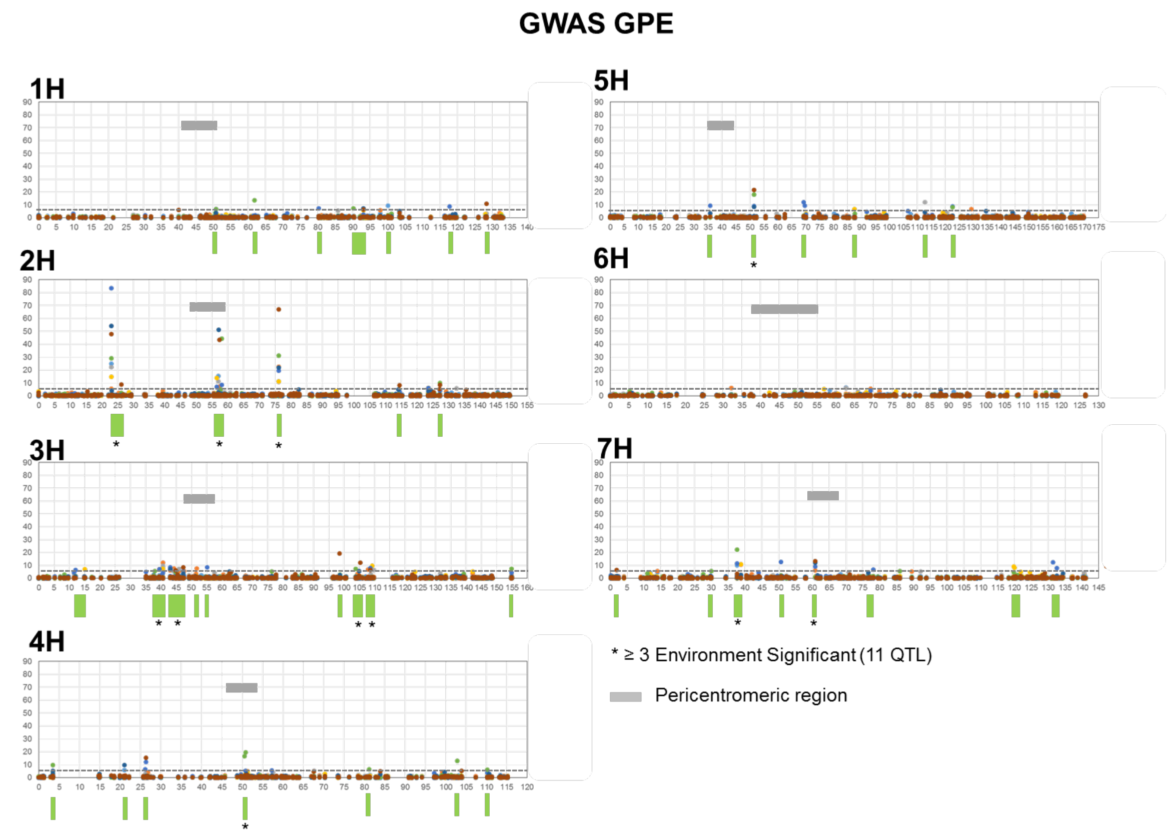

Figure S18: Distribution of grains per ear (GPE) QTLs across barley chromosomes.

GWAS GR

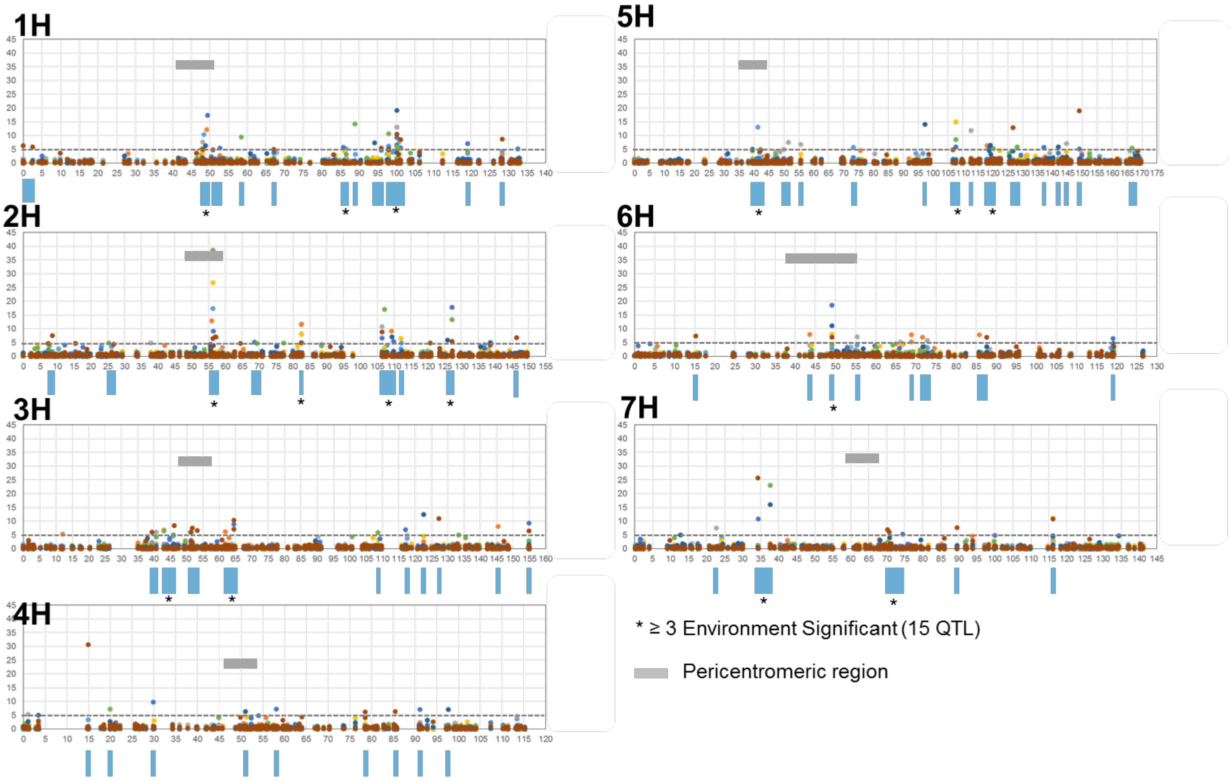

Figure S19: Distribution of grain roundness (GR) QTLs across barley chromosomes.

GWAS YLD “Halle”

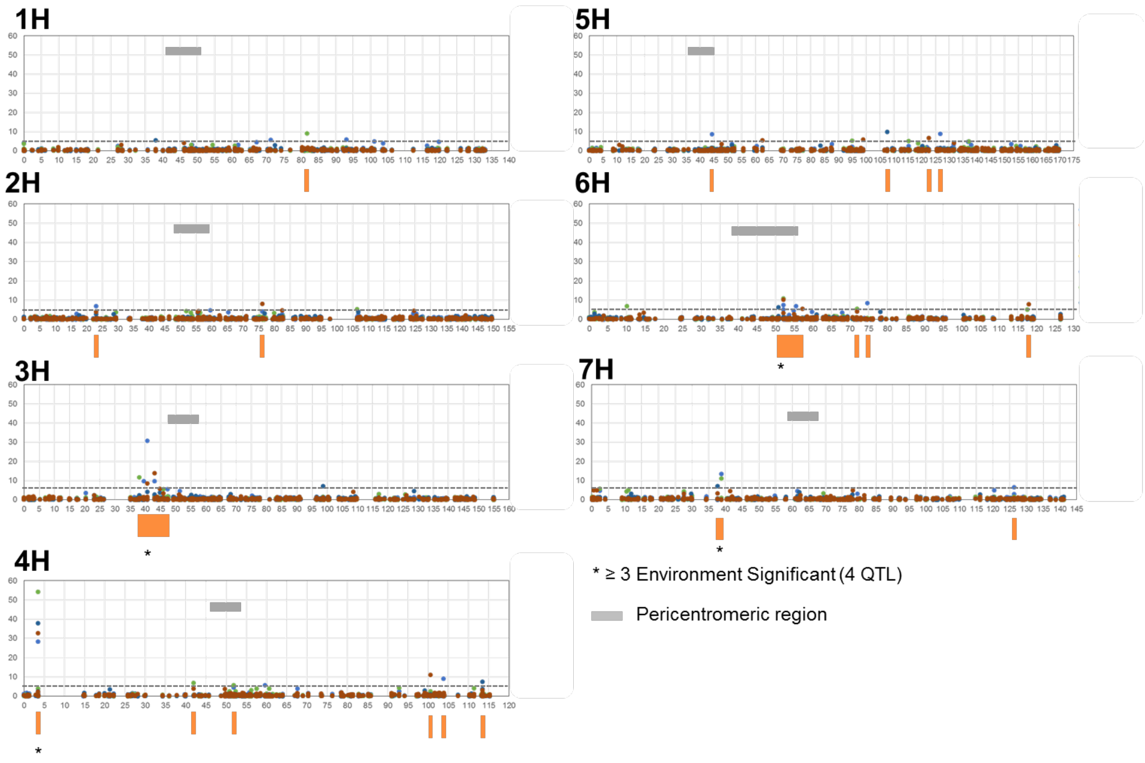

Figure S20: Distribution of grain yield (YLD) QTLs from Halle across barley chromosomes.

GWAS SE_GW

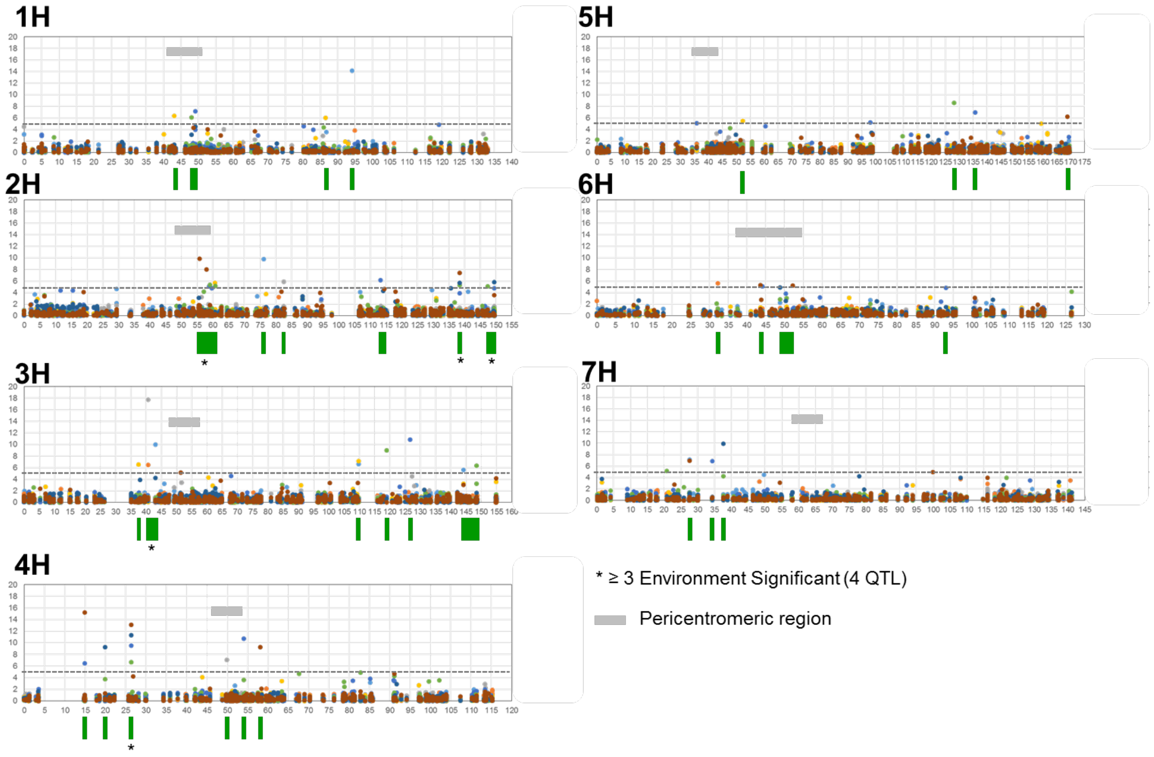

Figure S21: Distribution of standard error of grain width (SE_GW) QTLs across barley chromosomes.

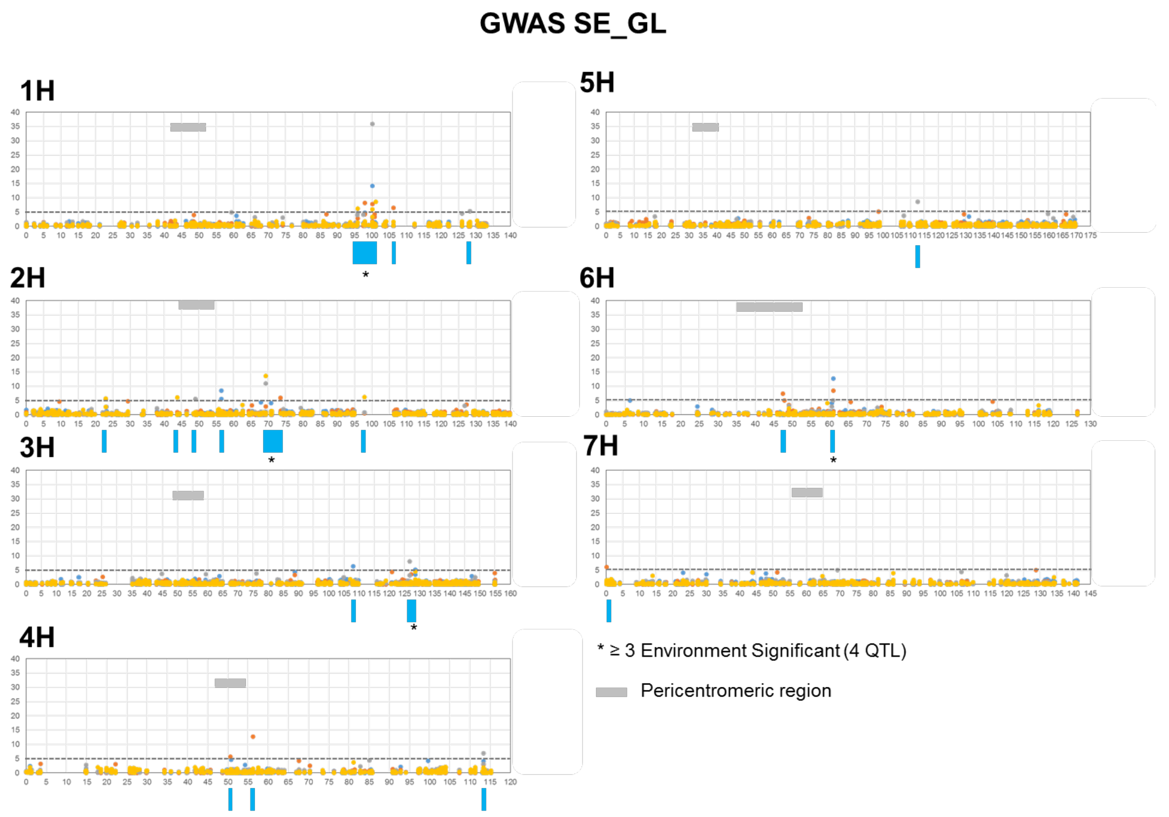

Figure S22: Distribution of standard error of grain length (SE_GL) QTLs across barley chromosomes.
